# Optimizing Reversible Phase‐Transformation of FeS_2_ Anode via Atomic‐Interface Engineering Toward Fast‐Charging Sodium Storage: Theoretical Predication and Experimental Validation

**DOI:** 10.1002/advs.202411884

**Published:** 2024-11-18

**Authors:** Wenxi Zhao, Yanbing Zhou, Hao Zhou, Xinqin Wang, Shengjun Sun, Xun He, Yongsong Luo, Binwu Ying, Yongchao Yao, Xiaoqing Ma, Xuping Sun

**Affiliations:** ^1^ School of Electronic Information Engineering Yangtze Normal University Fuling Chongqing 408100 China; ^2^ College of Chemistry, Chemical Engineering and Materials Science Shandong Normal University Jinan Shandong 250014 China; ^3^ Institute of Fundamental and Frontier Sciences University of Electronic Science and Technology of China Chengdu Sichuan 610054 China; ^4^ Department of Laboratory Medicine West China Hospital Sichuan University Chengdu Sichuan 610041 China; ^5^ School of Electronic Engineering Lanzhou City University Lanzhou Gansu 730070 China; ^6^ Center for High Altitude Medicine West China Hospital Sichuan University Chengdu Sichuan 610041 China

**Keywords:** anode, honeycomb‐like FeS_2_/SAs Mn@NC, reversible phase transformation, single‐atom catalyst, sodium‐ion batteries

## Abstract

Sodium‐storage performance of pyrite FeS_2_ is greatly improved by constructing various FeS_2_‐based nanostructures to optimize its ion‐transport kinetics and structural stability. However, less attention has been paid to rapid capacity degradation and electrode failure caused by the irreversible phase‐transition of intermediate Na_x_FeS_2_ to FeS_2_ and polysulfides dissolution upon cycling. Under the guidance of theoretical calculations, coupling FeS_2_ nanoparticles with honeycomb‐like nitrogen‐doped carbon (NC) nanosheet supported single‐atom manganese (SAs Mn) catalyst (FeS_2_/SAs Mn@NC) via atomic‐interface engineering is proposed to address above challenge. Systematic electrochemical analyses and theoretical results unveil that the functional integration of such two type components can significantly enhance ionic conductivity, accelerate charge transfer efficiency, and improve Na^+^‐adsorption capability. Particularly, SAs Mn@NC with Mn‐N_x_ coordination center can reduce the decomposition barrier of Na_2_S and Na_x_FeS_2_ to further accelerate reversible phase transformation of Fe/Na_2_S→NaFeS_2_→FeS_2_ and polysulfides decomposition. As predicted, such FeS_2_/SAs Mn@NC showcases outstanding rate capability and fascinating cyclic durability. A sequence of kinetic studies and ex situ characterizations provide the comprehensive understanding for ion‐transport kinetics and phase‐transformation process. Its practical use is further demonstrated in sodium‐ion full cell and capacitor with impressive electrochemical capability and excellent energy‐density output.

## Graphical abstract

Coupling FeS_2_ nanoparticles with honeycomb‐like nitrogen‐doped carbon nanosheet supported single‐atom manganese catalyst (FeS_2_/SAs Mn@NC) is proposed for the first time. Based on the remarkable advantages of ingenious nanoarchitectures, FeS_2_/SAs Mn@NC presents significantly improved phase‐transformation and admirable electrochemical performance.

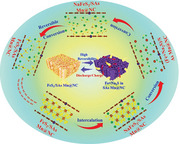

## 1 Introduction

As a fast‐charging electrochemical energy‐storage system, sodium ion batteries (SIBs) are regarded as the most appealing alternative solution to lithium‐ion batteries (LIBs) in that they possess significant advantages involving environmental friendliness, comparatively low price, and analogous electrochemistry.^[^ ^1^, ^2^, ^3^, ^4^, ^5^, ^6^ ^]^ In comparison to Li^+^, Na^+^ possesses a larger ionic radius and thus SIBs suffer from more severe issues of energy‐power tradeoff, slow reaction kinetics, and structural deformation than LIBs.^[^ ^7^, ^8^ ^]^ In this regard, it is imperative for elaborately exploring advanced anode materials to fit into the smooth Na^+^ accommodation whilst simultaneously showcasing high‐capacity and fast‐charging behaviors.

Pyrite ferric sulfide (FeS_2_), a plentiful and non‐toxic substance, is thought to be a viable candidate for conversion‐type anode materials, with state‐of‐the‐art characteristics of desirable theoretical capacity (894 mAh g^−1^), four‐electron redox reaction feature (FeS_2_ + 4 Na → Fe + 2 Na_2_S), and readily controllable architecture.^[^ ^9^, ^10^, ^11^, ^12^, ^13^ ^]^ The significant cycling ability and energy output for conversion‐type FeS_2_ anode largely depend on its high reaction reversibility and structural stability. Unfortunately, Na_x_FeS_2_ generated via the Na^+^‐intercalation reaction in the sodiation process cannot transform to pristine FeS_2_ after initial charging, resulting in low capacity of subsequent cycles.^[^ ^14^ ^]^ Moreover, the shuttling and dissolution of sodium polysulfides (NaPSs) also leads to rapid capacity degradation and final electrode failure.^[^ ^15^, ^16^, ^17^, ^18^ ^]^ Nanostructure engineering is proven as an effective strategy to enhance the structure stability and reaction kinetics of FeS_2_ anode.^[^ ^12^, ^19^, ^20^, ^21^ ^]^ Nevertheless, these FeS_2_‐based anodes are still challenged with material segregation, NaPSs shuttling, and irreversible conversion. Implanting rich catalytic sites into electrode materials could prominently improve reaction kinetics, thereby boosting reaction reversibility.^[^ ^22^ ^]^ Transition metal single‐atom (SAsM) nitrogen‐coordinated carbon‐based materials (SAsM‐NCCM) possess rich and uniform catalytic sites, high atom utilization efficiency, and strong adsorption capability which are advantageous for enhancing the electric conductivity, reaction kinetics, and electrochemical reversibility of the anode materials for SIBs.^[^ ^23^, ^24^, ^25^, ^26^, ^27^ ^]^ We, therefore, anticipate that the marriage of FeS_2_ with SAsM‐NCCM would provide us with a superb anode material for SIBs, which however remains unexplored so far.

In this work, we present a proof‐of‐concept demonstration of designing and constructing a honeycomb‐like hybrid of FeS_2_ nanoparticles coupled with N‐doped carbon (NC) nanosheet supported SAs Mn (FeS_2_/SAs Mn@NC) toward fast‐charging sodium storage. The SAs Mn@NC component directly tunes electronic structure, Na^+^‐adsorption capability, and electric conductivity of FeS_2_. Theoretical simulations reveal the great effect of SAs Mn@NC on improving the catalytic decomposition of Na_2_S and intermediate NaFeS_2_ phase during the charging process, enabling good reversible phase‐transformation of Fe/Na_2_S→NaFeS_2_→FeS_2_, confirmed by a series of ex situ electrochemical characterizations. Additionally, the interconnected conductive framework architecture of 3D honeycomb‐like NC nanosheet substantially shortens ion‐diffusion path and greatly reduces volume variation, guaranteeing stable cycle performance. Benefiting from these advantages, FeS_2_/SAs Mn@NC is superior in cycling endurance and high‐rate capability to most reported electrode materials. Our FeS_2_/SAs Mn@NC also shows great practical potential in sodium‐ion full cells and capacitors.

## 2 Results and Discussion

**Figure 1:**
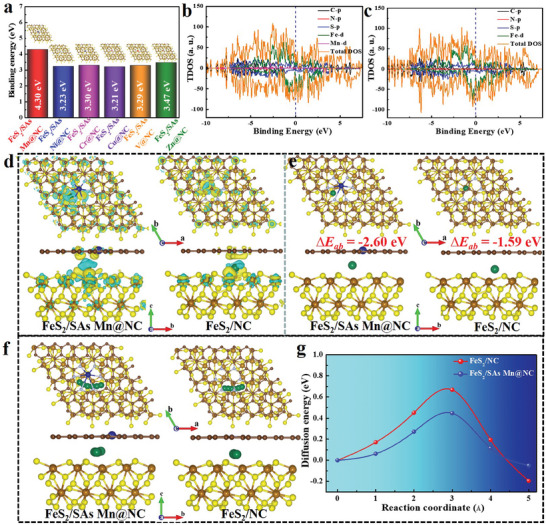
Theoretical prediction and analysis. a) Binding energies of FeS_2_ on SAs Mn@NC, SAs Ni@NC, SAs Cr@NC, SAs Cu@NC, SAs V@NC, and SAs Zn@NC. DOS of b) FeS_2_/SAs Mn@NC and c) FeS_2_/NC. d) Charge density differences and e) Na^+^‐adsorption energy of FeS_2_/SAs Mn@NC and FeS_2_/@NC. f) Na^+^‐diffusion paths and g) the corresponding energy barriers of FeS_2_/SAs Mn@NC and FeS_2_/@NC.

The most potential transition metal single atoms in the SAsM@NC catalysts were screened by employing theoretical simulations to further improve the electrochemical Na^+^‐storage capability of FeS_2_. First, several different heterostructure computational models were constructed (Figures S1 and S2, Supporting Information). Obviously, SAs Mn@NC was examined to possess the most remarkable interfacial binding energy with FeS_2_, reaching to 4.30 eV, among the six heterostructure models according to the adsorption energy (E_ad_) measurements (**Figure** **1**a), which demonstrates the strongest structural stability and best reaction kinetics in the discharging process.^[^ ^28^ ^]^ Moreover, the total density of states (DOS) of FeS_2_/SAs Mn@NC and FeS_2_/NC were performed to afford the compelling evidence for the enhanced electrical conductivity of FeS_2_ by the SAs Mn@NC (Figure 1b,c). According to the density differences at the Fermi level, both models are conductor with high DOS distributing, thus signifying high electronic conductivity. But FeS_2_/SAs Mn@NC model possesses a stronger electron delocalization near the Fermi level compared to FeS_2_/NC model due to the presence of SAs Mn in NC. That is, the Na^+^‐storage process on FeS_2_/SAs Mn@NC has more outstanding storage kinetics with better electronic conductivity. Further investigation about the charge density difference of optimized one Na atom inserted FeS_2_/SAs Mn@NC and FeS_2_/NC configurations (named as FeS_2_/SAs Mn@NC‐Na and FeS_2_/NC‐Na) were conducted to explore the influence on electronic transport (Figure 1d), where the electron gain and loss are respectively represented by yellow and cyan regions in the above models. The noticeable transfer of valence electrons in two models can be observed, with the Na atom consuming electrons while hybrid FeS_2_/SAs Mn@NC and FeS_2_/NC structures including carbon surface and FeS_2_ surface accumulating electrons. Meanwhile, compared to FeS_2_/NC (0.76 e^−^), FeS_2_/SAs Mn@NC model reveals a stronger electron transfer characteristic (0.78 e^−^) according to Bader charge analysis, meaning the existence of SAs Mn@NC is more favorable for trapping Na^+^, which could endow a higher redox reaction activity.^[^ ^29^ ^]^ Subsequently, we investigated in more detail the adsorption ability of two configurations for Na^+^ ion and the corresponding adsorption structural models are revealed in Figure 1e. Through calculation, FeS_2_/SAs Mn@NC model presents a greater negative adsorption energy (−2.60 eV) relative to FeS_2_/NC (−1.59 eV), thus symbolizing that the existence of SAs Mn in NC endows the electrode with more remarkable Na^+^‐adsorption ability, thereby contributing to richer active sites and faster reaction kinetics. Afterward, the energy barriers for Na^+^‐diffusion of both models along various diffusion states were further explored (Figure 1f). The Na^+^‐migration energy barriers of FeS_2_/SAs Mn@NC and FeS_2_/NC models are ≈0.45 and 0.67 eV (Figure 1g), respectively. The lower diffusion energy barrier of FeS_2_/SAs Mn@NC ensures better Na^+^‐diffusion kinetics and migration capability upon cycling, inducing superior rate capability. The aforementioned computation findings indicate that the introduction of SAs Mn in NC can effectively improve the electronic conductivity of FeS_2_, offer rich active sites, and be more conducive to Na^+^‐adsorption and structural stability.

**Figure 2:**
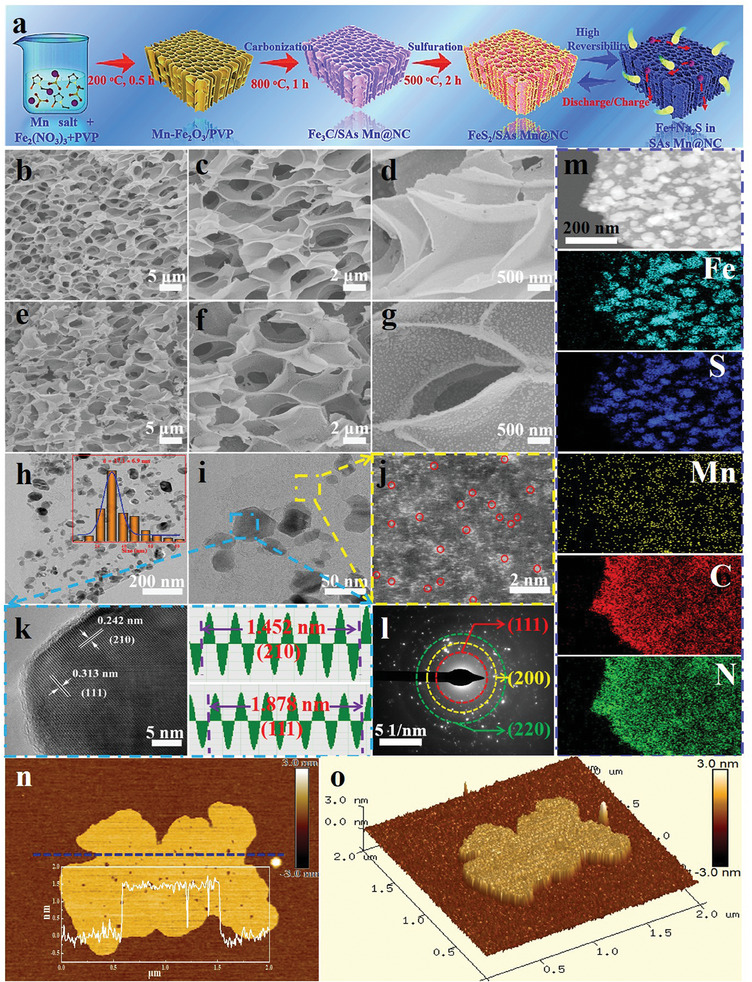
Structural characterizations. a) Schematic of preparation strategy for FeS_2_/SAs Mn@NC. FESEM images of the samples: b–d) FeC_3_/SAs Mn@NC, e–g) FeS_2_/SAs Mn@NC. h,i) TEM images, j) AC‐STEM‐ADF image, k) HRTEM image, l) SAED pattern, and m) HAADF‐STEM and corresponding EDX elemental mappings of FeS_2_/SAs Mn@NC. n,o) AFM images of FeS_2_/SAs Mn@NC. Inset in (h): the grain size profile of FeS_2_/SAs Mn@NC.

**Figure** **2**a provides a visual diagram for the whole fabrication technique of FeS_2_/SAs Mn@NC composites. Primarily, a conventional chemical foaming procedure was executed out making use of Fe[(NO_3_)]_3_·9H_2_O and polyvinyl pyrrolidone (PVP) as the foaming agent and carbon source, respectively, to generate 3D honeycomb‐like N‐doped carbon nanosheet architecture. As reaction temperature rises, melted PVP can be blown into a thin‐walled structure by NO_2_ gas produced by the breakdown of Fe[(NO_3_)]_3_·9H_2_O.^[^ ^30^, ^31^ ^]^ Following high‐temperature carbonization treatment, carbon reduces Fe_2_O_3_ to yield Fe_3_C, which inevitably consumes several of adjacent carbon,^[^ ^32^, ^33^ ^]^ thereby building a 3D honeycomb‐like porous architecture to implement isolated Mn atoms (denoted as Fe_3_C/SAs Mn@NC), and meanwhile the as‐formed Fe_3_C nanoparticles can be evenly confined into 3D architecture above. The morphological characteristics of the samples were evaluated through field‐emission scanning electron microscopy (FESEM) and transmission electron microscopy (TEM). As depicted in Figure 2b,c, PVP derived carbon manifests a highly regular three‐dimensional (3D) honeycomb‐like microporous architecture constructed by interconnected thin carbon walls. The magnified image exhibited in Figure 2d clearly indicates that honeycomb‐like carbon skeleton shows a surface composed of numerous extremely small nanoparticles. In addition, X‐ray diffraction (XRD) pattern of the sample in Figure S3 (Supporting Information) reveals a orthorhombic Fe_3_C phase (JCPDF # 85–1317) with a spacing group of Pnma(62). It is noted that without any other crystal phases belonging to metallic Mn or Mn compounds (e.g. MnO_2_, Mn_2_O_3_, etc.) were detected in Fe_3_C/SAs Mn@NC composites, further revealing that there is no Mn‐based crystalline phase. After further high‐temperature sulphuration procedure, the targeted FeS_2_/SAs Mn@NC composites were constructed, which commendably preserved 3D honeycomb‐like microporous architecture of Fe_3_C/SAs Mn@NC composites, without any local structural collapse (Figure 2e,f), showcasing its excellent structural stability. Further examination by magnified FESEM image shows a large quantity of FeS_2_ nanoparticles are steadily anchored on ultrathin carbon walls (Figure 2g), which can be further verified through TEM measurement with different magnifications (Figure 2h,i). Moreover, the particle size of FeS_2_ was statistically analyzed by means of TEM result of Figure 2h, manifesting an average particle size of ≈17.1 nm (inset of Figure 2h). It is anticipated that such 3D honeycomb‐like interconnected carbon skeleton with abundant pore structure and large specific surface area can effectively reduce Na^+^‐transfer resistance and offer fast ion/electron transport channel whilst enabling efficient electrolyte access into host materials.^[^ ^34^, ^35^ ^]^ Afterward, the ultrathin carbon nanosheet in local region of FeS_2_/SAs Mn@NC were characterized by aberration‐corrected high‐angle annular dark‐field scanning transmission electron microscopy (HAADF‐STEM). As illustrated in Figure 2j, a significant amount of atomically independent Mn species is explicitly distributed across the carbon substrate, as reflected by the plentiful isolated bright dots marked by dotted red circles. In addition, employing inductively coupled plasma atomic emission spectrometry (ICP‐AES), it was determined that the mass loading of SAs Mn species and main Fe element in FeS_2_/SAs Mn@NC composites were explored to be ≈2.21 and 35.4 wt.%, respectively. Further exploration for FeS_2_/SAs Mn@NC was determined via high‐resolution TEM (HRTEM) and selected area electron diffraction (SAED). The HRTEM result, depicted in Figure 2k, discloses two clear crystal lattices with the interplanar d‐spacings of 0.242 and 0.313 nm that are respectively identified to be the (210) and (111) planes of pyrite FeS_2_. The SAED pattern in Figure 2l reveals that 0.313, 0.271, and 0.192 nm of concentric diffraction rings from the inside to outside stand for the lattice planes of (111), (200), and (220) of FeS_2_, respectively, foreboding its multiphase nature. The composition of FeS_2_/SAs Mn@NC composites can be determined as being constituted of the expected Fe, S, C, N, and Mn elements by energy dispersed X‐ray (EDX) elemental mapping analysis, as illustrated in Figure 2m. More importantly, main Fe and S elements in FeS_2_/SAs Mn@NC composites present almost identical structure pattern of FeS_2_ nanoparticles, whilst SAs Mn species render similar morphological characteristics with the ultrathin carbon nanosheets, further suggesting the successful construction of FeS_2_/SAs Mn@NC composites. The thickness of carbon nanosheet in FeS_2_/SAs Mn@NC composites was further examined to be ≈1.5 nm through atomic force microscopy (Figure 2n,o), further confirming the ultrathin and graphene‐like architecture of carbon skeleton. In comparison, the contrasted FeS_2_/NC composites were constructed through employing FeC_3_/NC composites (Figure S4a,b, Supporting Information) as the precursor. As described in FESEM (Figure S4c,d, Supporting Information) and TEM (Figure S5, Supporting Information) images, the morphological properties of FeS_2_/NC composites are nearly identical to those of FeS_2_/SAs Mn@NC, further illustrating that the existence of SAs Mn species can not affect the structural characteristics of final FeS_2_/SAs Mn@NC products. In addition, the SEM images of FeS_2_/SAs Mn@NC‐0.025 (Figure S6a,b, Supporting Information) and FeS_2_/SAs Mn@NC‐0.05 (Figure S6c,d, Supporting Information) were tested. It is found that both materials maintain a 3D honeycomb‐like structure, and a large number of FeS_2_ nanoparticles were uniformly attached to the carbon skeleton, further indicating that SAs Mn loading would not affect the structural characteristics of final FeS_2_/SAs Mn@NC products.

**Figure 3:**
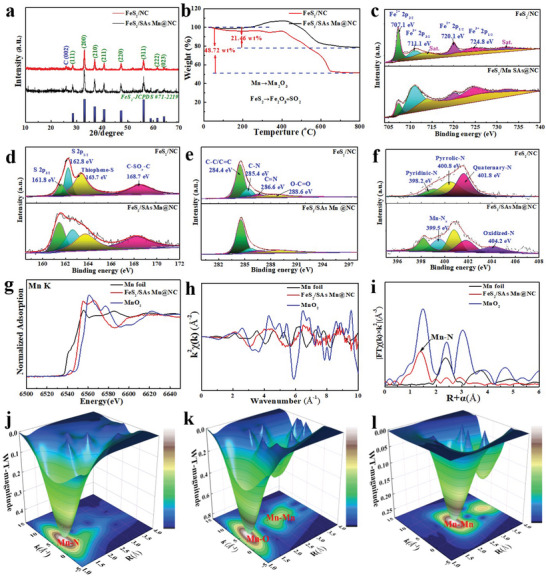
Phase structure, surface chemical constituents, and atomic environment characterizations. a) XRD patterns and b) TGA curves of FeS_2_/SAs Mn@NC and FeS_2_/NC. High‐resolution XPS spectra of FeS_2_/SAs Mn@NC and FeS_2_/NC: c) Fe 2p; d) S 2p; e) C 1s; f) N 1s. g) Mn K‐edge XANES, h) Fitted EXAFS, and i) FE‐EXAFS of FeS_2_/SAs Mn@NC, MnO_2_, and commercial Mn foil. WT contour plots at the Mn K‐edge for j) FeS2/SAs Mn@NC, k) MnO_2_, and l) commercial Mn foil.

XRD technique was used to identify the crystalline feature and phase composition of FeS_2_/SAs Mn@NC and FeS_2_/NC (**Figure** **3**a). It is evident that both samples manifest high crystallinity degree and same crystalline characteristics, and a series of reflection peaks, centered at 28.5°, 33.1°, 37.1°, 40.8°, 47.4°, and 56.3° assigning to the (111), (200), (210), (211), (220), and (311) crystal planes of cubic FeS_2_, are in match well with the standard card JCPDS# 71–2219 of FeS_2_ with spacing group of Pa‐3(205), indicating that SAs Mn species in FeS_2_/SAs Mn@NC composites haven't changed the phase composition of FeS_2_. In addition, a weak peak locates at ≈26.0° might be originated from NC skeleton. Thermogravimetric analysis (TGA) was conducted in air to ascertain the carbon contents in FeS_2_/SAs Mn@NC and FeS_2_/NC. As described in Figure 3b, the loss of surface adsorbed water caused slightly weight loss prior to 300 °C. Following that, FeS_2_ in the sample broke down between 300 and 650 °C to produce FeS and Fe_2_(SO_4_)_3_, which then turned into Fe_2_O_3_ and SO_2_.^[^ ^36^, ^37^ ^]^ In the meantime, NC and SAs Mn can be turned into CO_2_ and Mn_2_O_3_ within this temperature range.^[^ ^38^ ^]^ In addition, the XRD pattern of combustion products of FeS_2_/SAs Mn@NC was further conducted (Figure S7, Supporting Information), which involves bixbyite Mn_2_O_3_ (JCPDS #41‐1442) and hematite Fe_2_O_3_ (JCPDS # 33–0664). Notably, the mass increase in two samples in the range of 300–450 °C may be attributed to the oxidation of FeS_2_. Consequently, the mass proportions of NC in FeS_2_/SAs Mn@NC and FeS_2_/NC are close to 13.5 and 21.6 wt.%, respectively. From Raman spectra of two samples exhibited in Figure S8 (Supporting Information), two noticeable scattering shifts, situated at ≈1345.9 and 1584.2 cm^−1^, belong to D band resulting from disordered sp3‐type defective carbon and G band generated by the E_2g_ mode of crystalline graphitic carbon, respectively.^[^ ^11^, ^39^ ^]^ Moreover, in comparison to FeS_2_/NC (0.98), FeS_2_/SAs Mn@NC manifests a more significant I_D_/I_G_ ratio reaching to 1.02, signifying a greater amount of the structural defects’ density triggered by the combination of SAs Mn implanting and N‐doping in the FeS_2_/SAs Mn@NC. N_2_ adsorption–desorption isotherms were conducted to discover more about the porous features of two samples. As exhibited in Figure S9 (Supporting Information), FeS_2_/SAs Mn@NC presents a typical type IV isotherm with one discernible capillary condensation step and an H3 hysteresis loop^[^ ^37^ ^]^; and the corresponding Brunauer–Emmett–Teller (BET) surface area is ≈186.4 m^2^ g^−1^ with an average pore diameter of 6.7 nm, significantly greater than that of FeS_2_/NC (171.1 m^2^ g^−1^). A larger surface area of 3D honeycomb‐like architecture with rich mesoporous structure could not only offer sufficient contact area and ion‐transport channel at the electrode/electrolyte interface, yet might additionally enhance Na^+^‐ion migration and minimize Na^+^‐ion transfer resistance, these are beneficial for cycling properties.

X‐ray photoelectron spectroscopy (XPS) spectra of two samples was conducted to collect their surface chemical constituent and electronic structure. The full survey spectra reveal the co‐existence of involved Fe, S, C, N, and O elemental signals in two samples (Figure S10a, Supporting Information). In addition, a weak Mn 2p XPS peak was also detected in FeS_2_/SAs Mn@NC. The high‐resolution Fe 2p spectra in Figure 3c presents two pair reflection signals of Fe 2p_3/2_ and Fe 2p_1/2_, where two signal peaks at 707.1 and 720.1 eV can be regarded as Fe 2p_1/2_ and Fe 2p_3/2_ of Fe^2+^, respectively. While Fe 2p_3/2_ and Fe 2p_1/2_ of Fe^3+^ may be the main reason for the presence of two additional reflections, which are positioned at 711.1 and 724.8 eV, respectively.^[^ ^13^ ^]^ The high‐resolution S 2p spectra of two samples include four different bonding configurations (Figure 3d), which are responsible for S 2p_3/2_ and S 2p_1/2_ orbitals of divalent sulfur ions, thiophene‐S, and SO_x_ species at 161.8, 162.6, 163.7, and 168.6 eV, respectively.^[^ ^40^, ^41^ ^]^ As recorded in Figure 3e, the signals stemmed from C─C/C═C, C─N, C═N, and O─C═O bonds in high‐resolution C 1s spectra are situated at the binding energies of 284.4, 285.4, 286.6, and 288.6 eV, respectively.^[^ ^24^, ^42^ ^]^ The N 1s core level peak of FeS_2_/SAs Mn@NC can be divided into five bonding configurations (Figure 3f), involving pyridinic‐N (398.2 eV, 18.6%), Mn‐N*_x_* (399.5 eV, 20.9%), pyrrolic‐N (400.8 eV, 24.1%), quaternary‐N (401.8 eV, 17.7%), and oxidized N (404.2 eV, 18.7%).^[^ ^43^, ^44^ ^]^ In comparison to the N 1s spectrum of FeS_2_/NC, the proportion of Mn‐N*_x_* species increases from 0% to 10.9%, accompanied with a 8.2% reduction of pyridinic‐N in FeS_2_/SAs Mn@NC, thus suggesting that pyridinic‐N coordinates with Mn atoms more readily to yield Mn‐N*_x_* moieties.^[^ ^38^, ^45^ ^]^ The deconvolution of Mn 2p spectrum (Figure S10b, Supporting Information) is made up of two characteristic peaks positioned at 641.5 and 653.4 eV for Mn 2p_3/2_ and Mn 2p_1/2_, respectively, where the Mn 2p_3/2_ peak of FeS_2_/SAs Mn@NC is near to Mn^2+^, suggesting that the majority of SAs Mn in FeS_2_/SAs Mn@NC exists two valent Mn species.^[^ ^38^, ^46^ ^]^ X‐ray absorption near‐edge structure (XANES) and extended X‐ray absorption fine structure (EXAFS) spectroscopy at the Mn K‐edge were carried out to further evaluate the local electronic structure and coordination information of FeS_2_/SAs Mn@NC at the atomic level. For Mn K‐edge XANES (Figure 3g), the absorption edge position of FeS_2_/SAs Mn@NC is situated between Mn foil and MnO_2_, further indicating the presence of an intermediate oxidation state between Mn^0^ and Mn^4+^ for the isolated Mn atom in FeS_2_/SAs Mn@NC, which agrees well with the XPS result, based on the existence of particular shake‐up satellite peak at 646.2 eV.^[^ ^46^, ^47^ ^]^ Meanwhile, the local atomic environment manifests a large difference between Mn K‐edge oscillation functions κ2χ(k) of FeS_2_/SAs Mn@NC and those of MnO_2_ and Mn foil (Figure 3h), which is further verified by the matched FT‐EXAFS spectra. As manifested in Fourier transform (FT) EXAFS plot of FeS_2_/SAs Mn@NC (Figure 3i), a single primary FT‐EXAFS peak appeared at 1.46 Å, which was different from the Mn‐Mn coordination peak (Mn foil) and Mn‐O coordination peak (MnO_2_), can be identified the Mn‐N scattering channel,^[^ ^46^ ^]^ and meanwhile without other coordination signal peaks associated with Mn‐Mn and Mn‐S scattering pathway were detected, suggesting the formation of atomically dispersed Mn species in the FeS_2_/SAs Mn@NC. The structural characteristics of FeS_2_/SAs Mn@NC was examined employing quantitative EXAFS curve fitting analysis (Figure S11 and Table S1, Supporting Information), and the best‐fitting analysis unequivocally presents that the coordination number of Mn─N bond is ≈5.6, and the average bond length reaches to 1.94 Å, indicating that the isolated Mn atoms are about sixfold coordinated by N atoms. In addition, the wavelet transform (WT) EXAFS studies with strong resolution were also carried out in the k and R spaces (Figure 3j–l). The WT results further reveal that, in comparison to Mn foil and MnO_2_, without any coordination information about Mn─Mn bond in FeS_2_/SAs Mn@NC was found, and FeS_2_/SAs Mn@NC presents the maximum scattering intensity at k ≈ 5.3 Å, attributable to the Mn─N coordination bond.

**Figure 4:**
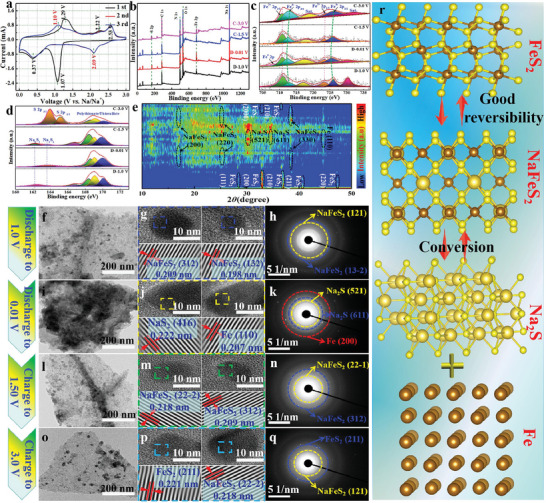
Reaction mechanism analysis. a) CV curves of FeS_2_/SAs Mn@NC at 0.5 mV s^−1^. Ex situ XPS spectra at various voltage states: b) XPS survey spectra, c) Fe 2p, d) S 2p. e) Ex situ 3D XRD contour map at different charge–discharge states. Ex situ TEM, HRTEM, and SAED at discharged states of f–h) 1.0 V, i–k) 0.01 V, and charged states of l–n) 1.50 V, o–q) 3.0 V. r) The graphical illustration of Na‐storage mechanism of FeS_2_/SAs Mn@NC.

The initial three cyclic voltammogram (CV) profiles at 0.5 mV s^−1^ were conducted to determine Na^+^‐storage capability and electrochemical reactivity for two FeS_2_‐based samples. As exhibited in **Figures** **4**a and S12 (Supporting Information), both samples manifest similar CV curve shape and redox peaks, demonstrating their similar electrochemical sodium‐storage mechanism. Taking FeS_2_/SAs Mn@NC for example, obviously, two reduction peaks are visible due to the sequential Na^+^ insertion into FeS_2_ during the first cathodic scan. The formation of Na_x_FeS_2_ is responsible for the reduction peak at 1.07 V, whereas the peak at 0.37 V is indicative of the conversion of Na_x_FeS_2_ to metallic Fe and Na_2_S.^[^ ^48^ ^]^ As the subsequent desodiation process, the anodic scanning curve presents three oxidation peaks, symbolizing a progressive desodiation process. Specifically, the peak at around 1.29 V should indeed be regarded as the re‐generation of Na_x_FeS_2_, and two additional oxidation reflection signals at ≈2.26 and 2.54 V signify the desodiation of Na_x_FeS_2_.^[^ ^49^, ^50^ ^]^ Meanwhile, comparing to the charging scan with FeS_2_/NC (Figure S12, Supporting Information), FeS_2_/SAs Mn@NC exhibits a larger intensity of anodic peak at 2.54 V, suggesting the enhanced electrochemical reversibility and reactivity brought by SAs Mn@NC. Afterward, the subsequent CV profiles are highly repeatable, revealing excellent electrochemical reversibility of FeS_2_/SAs Mn@NC. Ex situ XPS analysis was undertaken on FeS_2_/SAs Mn@NC at various discharge–charge depths with the goal to shed the conversion mechanism of Fe^2+^. It can be found that there are some related elements, such as Fe 2p, S 2p, Na 1s, O 1s, etc., were detected at different discharge–charge states, as manifested in the XPS survey spectra of Figure 4b. Obviously, the characteristic peak area of Fe^2+^ decreases as the discharge progresses from 1.0 to 0 V, accompanied with the emergency of a new characteristic diffraction peak of Fe^0^ at ≈707.2 eV at a fully discharged state of 0.01 V, adequately demonstrating the occurrence of conversion reaction. Subsequently, the peak area of Fe^2+^ gradually increases with the charging process, indicating a reversible conversion reaction of FeS_2_.^[^ ^51^ ^]^ In addition, the peak positions of 2p_3/2_ and 2p_1/2_ of Fe^3+^ slightly migrate to the low binding energy as the discharge progresses, and then upon charging gradually recover (Figure 4c), also attesting good reversible valence evolution of Fe 2p element.^[^ ^40^ ^]^ Ex situ S 2p spectra at the discharged states of 1.0 and 0.01 V together with the charged state of 1.5 V (Figure 4d) show two weak characteristic peaks at ≈162.3 and 163.6 eV symbolizing residual Na_2_S_2_ and Na_2_S_4_ in metastable polysulphides.^[^ ^52^ ^]^ As the charge voltage resets to 3.0 V, two obvious peak signals about S 2p_3/2_ and S 2p_1/2_ appear, which means the reversible conversion reaction of FeS_2_. Furthermore, a wide peak in the energy range of 166–172 eV signifies the presence of oxidized sulfur (polythionate and thiosulfate) which results from the occurrence of some side reactions (e.g. the formation of SEI layer).^[^ ^53^ ^]^

To gain deeper understanding toward the unique electrochemical reaction mechanism of FeS_2_/SAs Mn@NC, ex situ XRD measurement combined with ex situ HRTEM characterization were executed to provide evidence about the phase transformation of the electrode over the initial discharge–charge cycle. As illustrated in the ex situ XRD results (Figure 4e; Figure S13, Supporting Information), the characteristic peaks located at 28.5°, 33.1°, 37.1°, 40.8°, and 47.4°, belonging to the (111), (200), (210), (211), and (220) facets of cubic FeS_2_, can be obviously detected at the open voltage. With the occurrence of sodium intercalation reaction at a discharged state of 1.0 V, the reflection signals belonging to FeS_2_ disappear, followed by the emergence of byproduct NaFeS_2_ phase at 16.5°, 25.7°, and 38.9°, which can be indexed to the (200), (220), and (330) planes of NaFeS_2_ phase (JCPDF No. 34–0935). The peaks associated with NaFeS_2_ gradually vanish as the electrode voltage quickly drops to 0.01 V, and meanwhile some new peak signals at 30.7° and 34.7° gradually emerges that can be identified based on Na_2_S (JCPDF No. 47–0178). Synchronously, metallic Fe (JCPDF No. 87–0722) peak at 44.5° with (110) crystalline facet can be also detected, suggesting the conversion reaction of NaFeS_2_ phase, in accordance with the CV results. As the electrode subsequently charged to be ≈1.5 V, the characteristic peak intensity, which represent the Na_2_S and Fe, gradually diminishes, recombination with the reconstitution of NaFeS_2_ at 16.5°, 25.7°, and 38.9°, manifesting the plausible occurrence of reverse Na^+^ deintercalation. Lastly, as the charge depth increases to 3.0 V, except for the NaFeS_2_ peak appearing at the end of charging, the reappearance of FeS_2_ at 33.1° and 37.1° demonstrates that the existence of SAs Mn@NC in FeS_2_/SAs Mn@NC can significantly improve transition dynamical features of NaFeS_2_ to FeS_2_. Following that, ex situ TEM, HRTEM and SAED patterns of FeS_2_/SAs Mn@NC at four specific voltage states were investigated to further corroborate the phase evolution results and structural change characterization. As depicted in Figure 4f,i,l,o, the electrode at different states maintained the almost same morphological structure as the initial FeS_2_/SAs Mn@NC, without obvious agglomeration of nanoparticles, indicating superior structural stability of FeS_2_/SAs Mn@NC during the whole charge–discharge process. HRTEM image in Figure 4g at a discharged state of 1.0 V exhibits two spacing lattices of 0.209 and 0.198 nm, attributable to the (312) and (132) planes of byproduct NaFeS_2_ phase. Meanwhile, the typical diffraction rings of (121) and (13‐2) planes of NaFeS_2_ further signifies Na^+^ insertion process (Figure 4h), all of those agree well with the ex situ XRD characterization results. The HRTEM image of the electrode (0.01 V) is manifested in Figure 4j, in which the interplanar d‐spacings of 0.222 and 0.207 nm are categorized as the (416) plane of Na_2_S and (110) plane of metallic Fe, respectively, verifying the occurrence of conversion reaction. Concurrently, three diffraction rings, highlighted in Figure 4k, which represent to the Na_2_S (521) and (611) facets and Fe (200) crystal plane in the SAED pattern, supplies the substantial backing for the conversion reaction. After reversibly charging to 1.5 V, the lattice signals (Figure 4m) and diffraction rings (Figure 4n) of NaFeS_2_ emerged again, thus symbolizing the occurrence of reverse conversion reaction. Upon full charging to 3.0 V, two distinct nanocrystalline regions associated with NaFeS_2_ (22‐2) plane and FeS_2_ (211) phase can be clearly observed (Figure 4p). Meanwhile, SAED pattern (Figure 4q) further offered the diffraction signals of both NaFeS_2_ and FeS_2_, confirming the partial electrochemical reversibility of FeS_2_ at initial charging process due to the existence of SAs Mn@NC catalyst. Based on the above series of ex situ characterizations, the corresponding sodium storage mechanism of FeS_2_ can be vividly displayed in Figure 4r.

**Figure 5:**
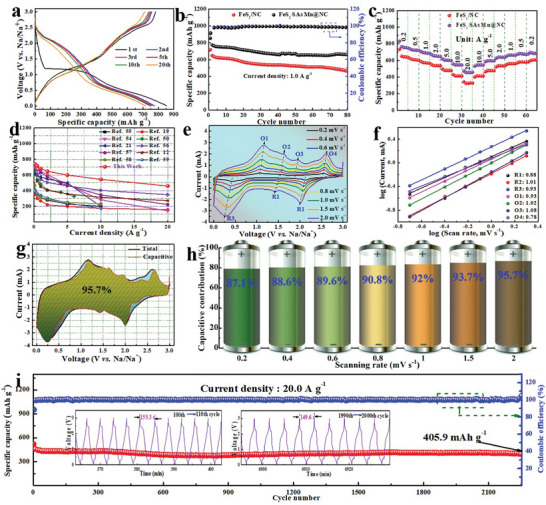
Electrochemical characterizations. a) GCD curves of FeS_2_/SAs Mn@NC at 1.0 A g^−1^. Comparison of b) cyclic lifespan and c) rate performance for FeS_2_/SAs Mn@NC and FeS_2_/NC. d) Capacity comparison of FeS_2_/SAs Mn@NC with other reported FeS_2_‐based anodes at different rates. e) CV curves at specific sweep speeds and f) b values determination at the marked peaks for FeS_2_/SAs Mn@NC. g) CV curve with the contribution percentage of FeS_2_/SAs Mn@NC at 2.0 mV s^−1^. h) Normalized contribution percentage of capacitively controlled charge from 0.2 to 2.0 mV s^−1^. i) High‐rate cycling durability of FeS_2_/SAs Mn@NC at 20.0 A g^−1^, inset in (i): the corresponding GCD profiles from 100th to 110th and 1990th to 2000th cycle.

Comparison on cycling stability and rate capability of two samples was further explored to clarify the significant effect on the reversible conversion and electrochemical Na^+^‐storage capability of FeS_2_ by the SAs Mn@NC catalyst. The initial galvanostatic charge–discharge (GCD) curves of FeS_2_/SAs Mn@NC and FeS_2_/NC at 1.0 A g^−1^ exhibit almost same redox voltage plateaus (**Figure** **5**a; Figure S15, Supporting Information), in good accordance with their CV results. Particularly, the initial discharge and charge capacities of FeS_2_/SAs Mn@NC electrode are recorded to be ≈854.8 and 782.9 mAh g^−1^, respectively, with a remarkable ICE reaching up to 91.6%, significantly exceeds to that of comparable FeS_2_/NC electrode (discharge/charge capacities of 727.0/ 652.3 mAh g^−1^ with 89.7% ICE). The reason for the higher ICE value of FeS_2_/SAs Mn@NC could be attributed to presence of SAs Mn@NC catalyst that accelerates high Na^+^ reversibility and prevents irreversible side reactions during the first sodiation/desodiation cycle. Furthermore, the GCD curves essentially overlap from the 2nd to 20th cycle, and meanwhile the corresponding CE value rises to 100% starting from the second cycle, further illustrating superior electrochemical reversibility. Following that, a further assessment on cycle stability of two samples was conducted at 1.0 A g^−1^ (Figure 5b). Impressively, FeS_2_/SAs Mn@NC achieves a higher discharge capacity reaching up to 658.5 mAh g^−1^ with a capacity retention of 77.1% over 80 cycles in comparison to its comparable electrode FeS_2_/NC, which only displays a 462.3 mAh g^−1^ capacity with a 64.4% retention. Meanwhile, the cyclic lifespan and specific capacity of NC was explored at same current rate and revealed in Figure S16 (Supporting Information), which only provide a reversible capacity of 150.0 mAh g^−1^ after 80 cycles. In addition, the GCD profiles and discharge capacities of FeS_2_/SAs Mn@NC‐0.025 and FeS_2_/SAs Mn@NC‐0.05 were also conducted at 1.0 A g^−1^ to further evaluate the effects of SAs Mn loading on the sodium‐storage performance of FeS_2_. Obviously, both samples show the similar GCD profiles (Figure S17, Supporting Information), indicating their same redox mechanism. Meanwhile, FeS_2_/SAs Mn@NC‐0.025 and FeS_2_/SAs Mn@NC‐0.05 electrodes provide the initial discharge capacities of 690.1 and 779.3 mAh g^−1^ at 1.0 A g^−1^, respectively. After 80 cycles, their capacities decrease to 574.1 and 601.1 mAh g^−1^, significantly less than that of FeS_2_/SAs Mn@NC (658.5 mAh g^−1^), further confirming that high SAs Mn loading on NC substrate can obviously improve the electrochemical performance of FeS_2_. To thoroughly comprehend exceptional electrochemical characteristics presented by FeS_2_/SAs Mn@NC, a comprehensive investigation of rate performance for two samples was performed (Figure 5c; Figure S18, Supporting Information). According to Figure 5c, at every current rate, FeS_2_/SAs Mn@NC demonstrates a significantly higher rate capacity when compared to FeS_2_/NC and pure NC (Figure S19, Supporting Information). FeS_2_/SAs Mn@NC provides an extraordinary discharge capability as high as 749.0, 719.5, 684.8, 648.3, 600.3, and 544.2 mAh g^−1^ at different rates by increasing the current density stepwise from 0.2 to 0.5, 1.0, 2.0, 5.0, and 10.0 A g^−1^. More interestingly, the electrode achieves an impressive reversible capacity of 459.5 mAh g^−1^ even after undergoing an extremely high current rate of up to 20 A g^−1^, which promptly regains a reversible capacity of 689.8 mAh g^−1^ and maintain a highly competitive retention up to 92.1% as the current rate gradually drops to initial 0.2 A g^−1^, revealing its outstanding structural durability and fast ion‐transport kinetics. Additionally, FeS_2_/SAs Mn@NC still exhibits greater electrochemical benefits when compared to earlier FeS_2_‐based reports.^[^ ^12^, ^19^, ^21^, ^50^, ^54^, ^55^, ^56^, ^57^, ^58^, ^59^ ^]^ Nyquist plots for two samples, highlighted in Figure S20a (Supporting Information), show that both are formed by making up of a straight line in the low frequency section that reflects the Warburg resistance (Z_w_), which is connected to Na^+^‐diffusion within the electrode, and a typical semicircle at the high‐middle frequency region that stands for the charge‐transfer impedance (R_ct_) on electrode/electrolyte interface. The R_ct_ value for FeS_2_/SAs Mn@NC was calculated to be ≈24.8 Ω (Table S3, Supporting Information), which is considerably smaller than that of FeS_2_/NC (60.9 Ω), reflecting its higher conductivity and faster charge‐transfer kinetics process. Additionally, the diffusion coefficient of Na^+^ ions (D_Na+_), one of the most significant parameters for the electrode, can be determined by exploiting the Z′ vs. ω^−1/2^ relationship in the low‐frequency Warburg region, the computed slope of which is inversely connected to the D_Na+_ value. FeS_2_/SAs Mn@NC electrode expresses a more impressive Na^+^ ion‐diffusion kinetics, as indicated in Figure S20b (Supporting Information), whereby its slope value is ≈20.3, significantly smaller than that of FeS_2_/NC electrode (34.9). Subsequently, EIS measurements were executed on FeS_2_/SAs Mn@NC following the 5th, 10th, 20th, and 30th cycles (Figure S21, Supporting Information). Obviously, it demonstrates relatively low R_ct_ value upon the whole cycling, as they reduce during the initial 5 cycles and marginally increase after 30 cycles, implying a good electrochemical contact between the electrode and electrolyte interface.^[^ ^60^ ^]^ Galvanostatic intermittent titration technique (GITT) curves of FeS_2_/SAs Mn@NC and FeS_2_/NC were obtained through experiencing a repeated 60 min pulse loop and 10 min relaxation at 0.1 A g^−1^ (Figure S22a, Supporting Information). The plots of the computed D_Na+_ against the state of charge in Figure S22c (Supporting Information) manifest that, in comparison to the contrast sample, FeS_2_/SAs Mn@NC possesses a significantly higher value of D_Na+_, disclosing significantly superior reaction kinetics. The enhanced capacitive contribution and rapid diffusion kinetics are probably related to the successful introduction of monatomic catalyst.

The reason for the outstanding cyclic capacity of FeS_2_/SAs Mn@NC, especially at high current rates, were explored by comparing pseudocapacitive contribution and electrochemical kinetic reaction features of both samples via CV curves at different scan rates. As seen in Figure 5e and Figure S23a (Supporting Information), the CV curves of two samples exhibit identical variation tendency with small peak shift as the scan rate increases, signifying a slight electrochemical polarization and excellent electrode's reversibility. In addition, the current response of FeS_2_/SAs Mn@NC is greater in comparison to that of FeS_2_/NC, signifying its stronger reaction capability. The power‐law relationship between peak current (*i*) and scan rate (*v*), in general, may be implemented to divide the total charge transfer process into diffusion‐controlled and faradaic reaction‐controlled processes:^[^ ^24^, ^39^ ^]^

(1) $$i=av^{b}$$

(2) logi=blogv+loga

where *a* is a variable parameter and *b* is a typical index that can be obtained from the slope by plotting log (*i*) vs log (*v*) to assess the reaction kinetics. Pseudocapacitive process is usually represented by a value of *b* near 1.0, whereas diffusion dominates the electrochemical process once the value is closer to 0.5.^[^ ^11^, ^61^ ^]^ As exhibited in Figure 5f, a series of *b*‐values can be fitted to 0.88, 1.01, 0.93, 0.93, 1.02, 1.08, and 0.78 at seven distinct redox peak current positions, respectively, based on a good linear relationship between logarithm peak currents and logarithm scan rates. The calculated *b*‐values are ≈1.0, implying that swift reaction‐controlled process accounts for the majority of total charge transfer. In addition, compared to FeS_2_/NC (0.92, Figure S23b, Supporting Information), a higher average *b* value (0.95) of FeS_2_/SAs Mn@NC stands out its more outstanding Faradaic reaction control process and rate capability. The kinetics characteristics of the samples for total charge storage were then conducted by using Equation *i* = k_1_ *v* + k_2_ *v* ^1/2^ to differentiate between two contribution behaviors, where k_1_ *v* and k_2_ *v* ^1/2^ stand for the contributions that are regulated by diffusion and capacitance, respectively. Not only FeS_2_/SAs Mn@NC offers an extraordinary pseudocapacitance contribution as high as 95.7% (yellow–green section in Figure 5g) at a scan rate of 2.0 mV s^−1^, but significantly superior to those of contrastive sample at all scan rates (Figure 5h; Figure S23d, Supporting Information), in consideration that superior conductivity and high surface area of FeS_2_/SAs Mn@NC accelerates swift electron transfer and offers abundant Na^+^ storage active sites. Surprisingly, FeS_2_/SAs Mn@NC manifests a surprising cycle performance, achieving a highly competitive reversible capacity of 405.9 mAh g^−1^ after 2200 cycles with an 84.0% capacity retention (relative to the second capacity) when operating at an ultrahigh current rate of 20.0 A g^−1^ (Figure 5i). Such superior Na^+^ storage performance also exceeds many reported FeS_2_‐based anodes (Table S2, Supporting Information). It is encouraging that, at such a high rate, the charge–discharge procedure could be finished within only 155.3 s during the first 110 cycles, which can be maintained at ≈149.6 s in a range of 1990th–2000th cycles. In the meantime, nearly identical GCD profiles reveal the steady phase evolution of FeS_2_/SAs Mn@NC throughout the continuous Na^+^ de‐/intercalation process.

**Figure 6:**
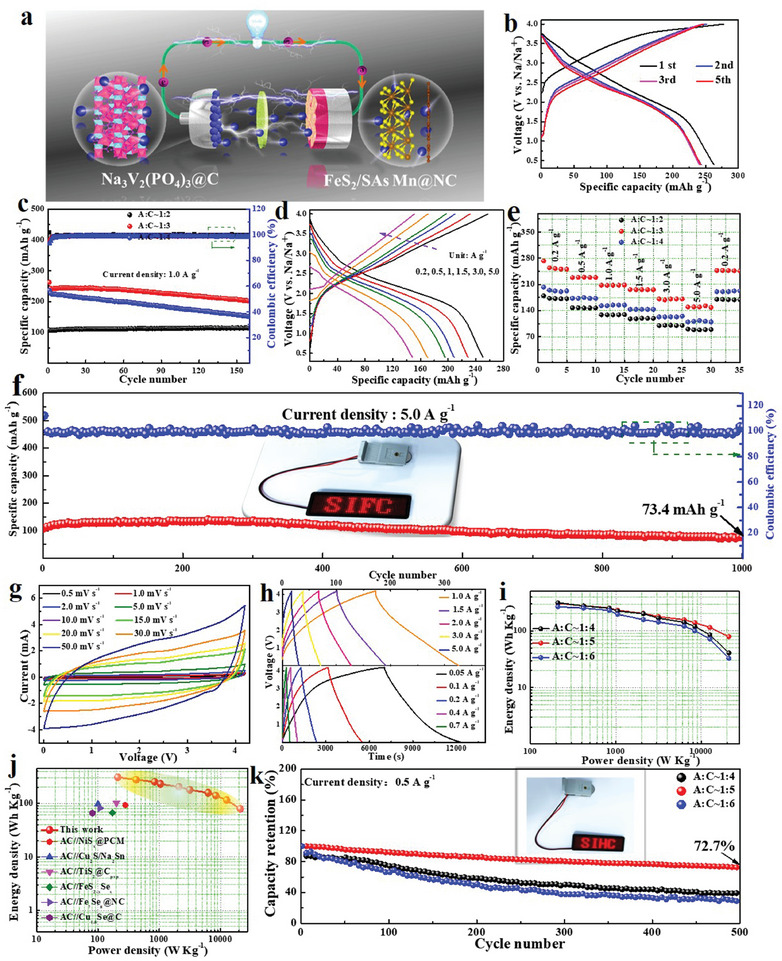
Evaluation of practical feasibility. a) Schematic illustration, b) initial five GCD profiles at 1.0 A g^−1^, and d) GCD curves at varied current rates of FeS_2_/SAs Mn@NC//NVP@C SIFCs. Comparison of c) cyclic stability and e) rate performance for FeS_2_/SAs Mn@NC//NVP@C SIFCs at different mass ratios of A:C. f) Cycling feature exploration of FeS_2_/SAs Mn@NC//NVP@C SIFCs at 5.0 A g^−1^. g) CV curves in the scan rate range of 0.5 to 50.0 mV s^−1^ and h) GCD profiles in the rate range of 0.05–5.0 A g^−1^ for FeS_2_/SAs Mn@NC//AC SIHCs. i) Ragone plots suggesting the energy and power densities at different mass ratios of A:C. j) Ragone plots based on the total mass of anode and cathode compared with those reported metal sulfide‐based SIHCs. k) Comparison of Cycling stability at different mass ratios of A:C at 0.5 A g^−1^.

To further estimate practical feasibility of FeS_2_/SAs Mn@NC on the energy‐storage fields, coin‐type sodium‐ion full cells (SIFCs) and hybrid capacitors (SIHCs) were manufactured employing preactivated FeS_2_/SAs Mn@NC as anode and commercial Na_3_V_2_(PO_4_)_3_@C (NVP@C) or activated carbon (AC) acting as cathode, and the diagrammatic drawing of SIFCs device during the charging‐discharging process is vividly illustrated in **Figure** **6**a. Note that these devices mentioned above can be named as FeS_2_/SAs Mn@NC//NVP@C SIFCs and FeS_2_/SAs Mn@NC//AC SIHCs for simplicity. First, XRD pattern and electrochemical performance of NVP@C and AC cathode were explored and exhibited in Figures S24–S26 (Supporting Information), respectively, both of which can offer excellent cyclic performance over 200 cycles with high reversible capacity in a half cell configuration. To optimize the electrochemical performance of SIFCs, the mass ratios between FeS_2_/SAs Mn@NC anode and NVP@C cathode (denoted as A:C) were explored in the range from 1:2 to 1:4. The electrochemical GCD profiles of all assembled SIFCs devices at various mass ratio of A:C were revealed in Figure 6b and Figure S27 (Supporting Information). SIFCs in a mass ratio of A:C ≈1:3 offer initial discharge and charge capacities of 262.5 and 277.3 mAh g^−1^ at 1.0 A g^−1^ (Figure 6b), respectively, with a capacity retention over 94.7%, significantly exceeds to additional two SIHCs devices at the mass ratios of A:C ≈1:2 and 1:4. Meanwhile, starting from the 2nd cycle, the GCD curves of SIFCs basically overlap, manifesting the satisfactory reversibility of the composite. As the discharge–charge process continues to 160th cycle, SIFCs device with a mass ratio of A:C ≈1:3 achieves an outstanding reversible discharge capacity reaching to 202.2 mAh g^−1^ with a 98.9% CE value, while other two devices only deliver the discharge capacities of 152.2 and 113.8 mAh g^−1^ as the mass ratios of A:C are ≈1:4 and 1:2, respectively, suggesting that the optimal mass ratio of A:C is ≈1:3. Subsequently, the rate capabilities of SIFCs devices at different mass ratios of A:C were also explored. According to the GCD profiles of SIFCs at three mass ratios of A:C (Figure 6d; Figure S28, Supporting Information), SIFCs at a mass ratio of A:C ≈1:3 exhibits smaller voltage polarization especially at 3.0 and 5.0 A g^−1^ compared with other two kinds of mass ratios. Particularly, at a mass ratio of A:C ≈1:3, SIFCs demonstrates best rate capability amongst three kinds of mass ratios, delivering the reversible capacities of 250.3, 228.5, 208.3, 196.0, 170.9, and 148.3 mAh g^−1^ with the current rate rising from 0.2 to 0.5, 1.0, 1.5, 3.0, and 5.0 A g^−1^. Following that, as the current rate resets to original 0.2 A g^−1^, the specific capacity can still recover to 245.9 mAh g^−1^, with an excellent capacity retention of ≈98.2%, indicative of its extraordinary rate capability. More importantly, SIFCs at a mass ratio of A:C ≈1:3 acquire an ultralong cyclic lifespan over 1000 cycles at 5.0 A g^−1^ (Figure 6f), affording a reversible capacity of 73.4 mAh g^−1^ with a small capacity fade of only 0.03% per cycle, suggesting its outstanding cycling stability. Notably, an LED screen bearing the phrase “SIFC” (inset of Figure 6f) could potentially be successfully illuminated by the assembled coin‐type SIFCs, highlighting the high potential for practical use.

Following that, a FeS_2_/SAs Mn@NC//AC SIHCs device was constructed to further reveal the practical value of FeS_2_/SAs Mn@NC. Given that the capacitive cathode and battery‐typed anode have distinct energy‐storage methods, the mass ratio of FeS_2_/SAs Mn@NC anode and activated carbon cathode (A:C) can be adjusted to balance charge transport kinetics. Figure 6g and Figure S29 (Supporting Information) manifest the electrochemical CV features of all assembled SIHCs devices at different mass ratios of A:C. The multiple energy storage mechanisms in all assembled SIHCs devices can be confirmed by the “capacitor‐like” CV profiles deviating from the desirable rectangle shape.^[^ ^61^, ^62^ ^]^ Furthermore, at all scan rates, the outlines of CV profiles basically preserve their original shape without suffering from obvious deformation, demonstrating impressive reversibility and exceptional rate characteristic. Meanwhile, the quasi‐triangular features of GCD curves for SIHCs (Figure 6h; Figure S30, Supporting Information), which is similar to the ideal supercapacitor with the linear slope, are presented at varying rates due to the coexistence of the Faradaic and non‐Faradic processes.^[^ ^11^, ^39^, ^63^ ^]^ Subsequently, Ragone plots of SIFCs devices illustrating the energy and power densities at different mass ratios of A:C reveal that the optimal mass ratio of A:C is 1:5 (Figure 6i). In this configuration, SIFCs offer a maximum energy density output reaching to 308.6 Wh kg^−1^ at a power density of 210 W kg^−1^ and further sustained an outstanding energy density of 79.3 Wh kg^−1^ even at a highly competitive power density of 21 000 W kg^−1^, with a very short average charge–discharge time of only 30.7 s, suggesting excellent rate capability of SIHCs. Ragone plot gives additional evidence that the energy and power densities of SIFCs at a mass ratio of A:C≈1:5 are superior to numerous of state‐of‐the‐art SIHCs described in earlier research (Figure 6j).^[^ ^64^, ^65^, ^66^, ^67^, ^68^, ^69^ ^]^ In addition, the cycling ability of all assembled SIHCs devices at different mass ratios of A:C was further compared. As revealed in Figure 6k, SIHCs device with a mass ratio of A:C ≈1:5 exhibits good cycling stability with a 72.7% retention of the initial capacity after 500 cycles at 0.5 A g^−1^, superior to that of other two SIHCs devices. Meanwhile, an LED screen featuring “SIHC” typeface was successfully powered up (inset of Figure 6k), showcasing the great potential applications of FeS_2_/SAs Mn@NC in large‐scale energy storage systems.

**Figure 7:**
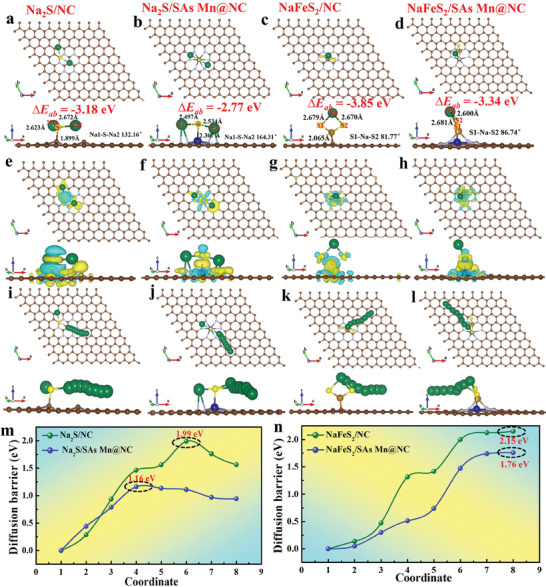
Mechanism insights. a–d) Atomic geometrical configurations and corresponding bond length and bond angle of Na_2_S and NaFeS_2_ adsorbed on NC and SAs Mn@NC substrates. e–h) Charge density differences of Na_2_S and NaFeS_2_ adsorbed on NC and SAs Mn@NC substrates. i–l) The detailed decomposition pathways of Na_2_S and NaFeS_2_ on NC and SAs Mn@NC substrates. The decomposition barriers of m) Na_2_S and n) NaFeS_2_ on NC and SAs Mn@NC substrate.

To better compare the catalytic activity of SAs Mn@NC and NC to Na_2_S and NaFeS_2_ during the first stage of Fe/Na_2_S→NaFeS_2_ and subsequent conversion step of NaFeS_2_→FeS_2_, Na_2_S and NaFeS_2_ molecules were firmly adsorded on the surface of SAs Mn@NC and NC substrates, respectively, and the corresponding geometrical configurations and electronic structures of Na_2_S/NC, Na_2_S/SAs Mn@NC, NaFeS_2_/NC, and NaFeS_2_/SAs Mn@NC models were exhibited in **Figure** **7**a–d. First, we analyzed the bond strength of Na─S in Na_2_S/NC and Na_2_S/SAs Mn@NC models to evaluate the catalytic activity of SAs Mn@NC and NC catalysts, of which the Na─S bond strength can be reflected by bond angle of Na1─S─Na2. Clearly seen that the Na1─S─Na2 angle on SAs Mn@NC substrate is ≈164.31°, greater than that on NC substrate (132.16°). Analogously, NaFeS_2_ on SAs Mn@NC substrate also possesses a larger S1‐Na‐S2 angle of 86.74° compared to that on NC substrate (81.77°), signifying that Na_2_S and NaFeS_2_ decompose more readily on SAs Mn@NC substrate.^[^ ^70^ ^]^ Note that Na_2_S on the NC substrate manifests the larger Na1─S bond of 2.62 Å and Na2─S bond of 2.67 Å relative to SAs Mn@NC substrate, meanwhile, a higher Na_2_S adsorption energy on NC (3.18 eV) signifies that the strong interaction makes it difficult to completely eradicate Na atom from NC catalyst, which negatively impacts the activity of NC catalyst and prevents the reaction that follows.^[^ ^17^ ^]^ Additionally, the charge density differences of four models in Figure 7e–h reveal that the Mn─S bonds in Na_2_S/SAs Mn@NC and NaFeS_2_/SAs Mn@NC models present significant charge accumulation regions, standing for a strong interaction between SAs Mn@NC and Na_2_S/NaFeS_2_. Following that, the decomposition barriers and detailed decomposition pathways of Na_2_S on NC and SAs Mn@NC substrates are presented in Figure 7i,j,m. It's worth noting that the decomposition process of Na2 atom involves two key steps: the breakage of the Na2─S bond and the removal of Na atoms. As presented in Figure 7m, due to the overlarge adsorption of Na_2_S on NC substrate, the Na_2_S decomposition energy barrier on NC substrate is as high as 1.99 eV, while the that of SAs Mn@NC substrate is only 1.16 eV, demonstrating that SAs Mn@NC with a lower decomposition energy barrier reveals its better catalytic activity for the decomposition of Na_2_S and reversible conversion reaction of first stage of Fe/Na_2_S→NaFeS_2_. More importantly, the catalytic effect of NC and SAs Mn@NC on the subsequent reaction of NaFeS_2_→FeS_2_ was further explored. Likewise, SAs Mn@NC showcases a high NaFeS_2_ adsorption energy of −3.34 eV, whilst NC achieves an excessively substantial value of −3.85 eV, which somewhat favors the formation of the Na─S bond.^[^ ^17^ ^]^ Furthermore, the decomposition barriers of NaFeS_2_ on two substrates were also studied. As revealed in Figure 7k,l,n, in comparison to NC substrate (2.15 eV), the decomposition barrier of NaFeS_2_ on SAs Mn@NC substrate is only 1.76 eV, symbolizing the better catalytic activity of SAs Mn@NC on reversible conversion reaction of NaFeS_2_→FeS_2_. Above these theoretical results confirm that SAs Mn@NC substrate exhibits superior catalytic activity for the pivotal step of Na_2_S and NaFeS_2_ decomposition, thereby accelerating the reversible electrochemical reaction of Fe/Na_2_S→NaFeS_2_→FeS_2_.

## 3 Conclusion

In summary, we present a proof‐of‐concept demonstration of designing and constructing a honeycomb‐like hybrid of FeS_2_ nanoparticles coupled with N‐doped carbon (NC) nanosheet supported SAs Mn (FeS_2_/SAs Mn@NC) via atomic‐interface engineering. The significant catalytic function of SAs Mn@NC component significantly tunes electronic structure, Na^+^‐adsorption capability, and electric conductivity of FeS_2,_ while facilitating NaPSs decomposition and good reaction reversibility during the initial charge process. Moreover, the unique architecture of FeS_2_/SAs Mn@NC endows favorable features with highly cross‐linked conductive network and abundant Na^+^‐storage active sites, enabling its superior battery performance. As a result, such FeS_2_/SAs Mn@NC harvests outstanding rate capability and durable cyclic lifespan over 2200 cycles with a competitive reversible capacity of 405.9 mAh g^−1^ at 20.0 A g^−1^. Additionally, a series of ex situ characteristic studies and theoretical calculations demonstrate the significant effect of SAs Mn@NC catalyst on improving NaPSs decomposition and reaction reversibility of FeS_2_. Our FeS_2_/SAs Mn@NC also demonstrates practical application potential in sodium‐ion full cells and capacitors, which manifests high‐capacity contribution, superior cycling performance, and attractive energy density output. This study not only strengthens the comprehensive understanding of electronic structure optimization strategy but provides a guide to design nanohybrids materials for high‐performance energy storage devices.

## Conflict of Interest

The authors declare no conflict of interest.

## Supporting information



Supporting Information

## Data Availability

The data that support the findings of this study are available from the corresponding author upon reasonable request.
