# Supplementary material for: Optimizing Reversible Phase‐Transformation of FeS2 Anode via Atomic‐Interface Engineering Toward Fast‐Charging Sodium Storage: Theoretical Predication and Experimental Validation
Source: Adv Sci (Weinh). 2024 Nov 18;12(2):2411884. doi: 10.1002/advs.202411884 (PMC11727254; doi:10.1002/advs.202411884)
Supplement: Supplementary file 1 — Supporting Information [file ADVS-12-2411884-s001.docx]

**Supporting Information**

**Optimizing Reversible Phase-Transformation of FeS_2_ Anode via Atomic-Interface Engineering toward Fast-Charging Sodium Storage: Theoretical Predication and Experimental Validation**

Wenxi Zhao,^†^ Yanbing Zhou,^†^ Hao Zhou, Xinqin Wang, Shengjun Sun, Xun He, Yongsong Luo, Binwu Ying, Yongchao Yao,* Xiaoqing Ma,* Xuping Sun*

**Experimental section**

**Synthesis of Fe3C coupled honeycomb-like N-doped carbon (NC) supported SAs Mn (FeC_3_/SAs Mn@NC).**

In a typical synthesis, 3.0 g of polyvinyl pyrrolidone (PVP, K30), 4.5 g of Fe(NO_3_)_3_·9H_2_O, and 0.075 g Mn(CH_3_COO)_2_·4H_2_O were successively added into 45 mL of deionized water while being stirred and ultrasonicated to form clear reddish-brown solution. Afterward, the mixture solution above was then completely dried at 60 °C, and the resulting yellow-brown powder was further grinded for about 30 min to form a homogenous precursor. Finally, the yellow-brown powder was subjected to heating at a rate of 5 °C min^-1^ under Ar for 0.5 h at 200 °C and then was carbonized for 1 h at 800 °C to yield the FeC_3_/SAs Mn@NC precursor. FeC_3_/NC was synthesized utilizing the same condition without adding Mn(CH_3_COO)_2_·4H_2_O. In addition, we further regulated the mass loading of SAs Mn on NC substrate by changing the addition amount (0.025 g and 0.050 g) of Mn(CH_3_COO)_2_·4H_2_O during the material preparation process to construct different FeS_2_-based materials, named as the FeS_2_/SAs [Mn@NC-0.025](mailto:Mn@NC-0.025) and FeS_2_/SAs [Mn@NC-0.05](mailto:Mn@NC-0.025), respectively.

**Synthesis of FeS_2_ coupled honeycomb-like N-doped carbon (NC) supported SAs Mn (FeS_2_/SAs Mn@NC).**

As-prepared 0.1 g of FeC_3_/SAs Mn@NC (or FeC_3_/NC) and 0.5 g of sublimed sulfur were added to two different quartz boats, which can be further wrapped together using aluminum foil. Then, two encapsulated porcelain boats were subsequently transferred to a tube furnace and further vulcanized at 500 ^o^C for 2 h under Ar flow with a heating rate of 2 °C min^-1^. After cooled to room temperature, the constructed samples were denoted as FeS_2_/SAs Mn@NC (or FeS_2_/NC). In addition, pure polyvinyl pyrrolidone derived NC can be also obtained at same carbonization temperature with FeS_2_/NC.

**Materials characterization.**

The morphological and microstructural characteristics, surface roughness, crystalline structure, specific surface area and pore size distribution, as well as disordered properties of the obtained products were characterized by field emission scanning electron microscopy (FESEM, XL30 ESEM FEG), transmission electron microscope (TEM, HRTEM, FEI Talos F200S), atomic force microscopy (AFM, Bruker Dimension Icon), X-ray diffraction (XRD, Shimadzu XRD-7000 X-ray diffractometer), Automated gas sorption analyzer (Micromeritics ASAP 2460), and Micro-Raman spectrometer (WITec alpha300R), respectively. The elemental components and surface valence states were evaluated employing Thermo Scientific K-Alpha X-ray photoelectron spectroscopy (XPS). The carbon content and loading capacity of Mn single atom in composites were characterized by TGA (Mettler-Toledo TGA/DSC 3+) and Inductively coupled plasma optical emission spectrometry (ICP-OES, Agilent 5110). Aberration-corrected high-angle annular darkfield scanning transmission electron microscope (HAADF-STEM) was collected on a FEI Titan G2 60-300. EXAFS measurements were performed at X-ray nanodiffraction (XND) beamline BL1606 by Canadian Light Source (CLS).

**Electrochemical performance evaluation of half cells.**

The CR2025-type coin cells, which were manufactured in a glove box with an Ar environment, were implemented to test all the electrochemical tests. Active material, carbon black, and sodium carboxymethyl cellulose (CMC) were thoroughly blended in the proper volume of water at a mass ratio of 7:2:1 to construct working electrodes. The resulting slurry was subsequently poured onto copper foil and vacuum-dried for 12 h at 60 °C. The mass loading of approximately 0.8~1.2 mg cm^-2^ were painted on each electrode. The counter electrode was manufactured employing handmade sodium foil with a diameter of roughly 16 mm, which purchased from Sinopharm Chemical Reagent Co., Ltd.. 1.0 M NaCF_3_SO_3_ in diethylene glycol dimethyl and glass fiber (Whatman, GF/D) that were employed in all electrochemical investigations were used as the electrolyte and separator, respectively. Over a voltage window of 0.01~3.0 V, the galvanostatic charge and discharge (GCD) cycles and cycling performance were examined making use of the Land CT3001A battery testing equipment. The specific capacity is obtained according to the weight of active materials on the electrode. The cyclic voltammetry (CV, cut-off voltage of 0 ~3.0 V) and electrochemical impedance spectroscopy (EIS, frequency range of 100 kHz ~0.01 Hz) were performed through an electrochemical workstation (CHI 660E). Galvanostatic intermittent titration technique (GITT), which applies pulse current of 0.1 A g^-1^ for 10 min, followed by 1 h relaxation, is being utilized to capture the important information stemmed from Na^+^-diffusion coefficients of the electrode. For ex-situ XRD test, the electrode was sodiated and desodiated at different voltage states at 0.05 A g^-1^. And then the cycled electrode at every specific voltage state was disassembled in an Ar-filled glove box and washed with diglyme several times before testing. Afterward, the disassembled electrode can be naturally dried in Ar atmosphere, which can be quickly transferred to XRD equipment to collect phase information.

**Electrochemical evaluation of sodium-ion full cells (SIFCs) and hybrid capacitors (SIHCs).**

Primarily, sodium-ion full cell and hybrid capacitor were manufactured in the CR2025 coin-type cell applying the same electrolyte and separator of the half-cell. Na_3_V_2_(PO_4_)_3_@C (NVP@C) or activated carbon (AC) (80 wt%), Super P (10 wt%), and polyvinylidene fluoride (PVDF, 10 wt%) were combined with N-methyl 2-pyrrolidene (NMP) to form a slurry, which was then coated on commercial aluminum foil and further dried at 100 °C overnight in a vacuum to create the cathode electrode. Prior to fabrication, FeS_2_/Mn SAs@NC anode was pre-activated for three cycles at 0.05 A g^-1^ in a half cell between 0.01 and 3.0 V versus Na/Na^+^, and then terminated at 0.01 V versus Na/Na^+^ so as to minimize irreversible capacity loss and stabilize the electrode surface. Note that, during the performance tests of full cell and hybrid capacitor, the mass loading of active material in the positive and negative electrodes is about 2.0 and 1.0 mg cm^-2^, respectively. Besides, the capacity ratio between positive and negative electrodes can be controlled in a range from 1:4 to 1:6. The different mass ratios between preactivated FeS_2_/Mn SAs@NC anode and NVP@C (or AC) cathode were explore to further optimize the electrochemical performance of SIFC and SIHC. In addition, both AC and NVP@C were originated from Guangdong Canrd New Energy Technology Co., Ltd. For cathode electrode, the mass loading density of active material is around 2.0 mg cm^-2^. For both SIFC and SIHC, the cut-off voltage windows were set in the potential ranges of 0.4~3.8 V for SIFC and 0.01~ 4.2 V for SIHC. The specific capacity of SIFC was calculated based on the anode's mass, while the energy (E, Wh kg^-1^) and power densities (P, W kg^-1^) of SIHC was obtained through the whole mass of anode and cathode according to the following equations:

P = ΔV × i/m (1)

E = P × t/3600 (2)

ΔV = V_max_ - V_min_ (3)

where m (Kg) is the total mass of active materials in the anode and cathode, Vmax (V) stands for the discharge potential excluding the IR drop, and Vmin (V) is the final discharge voltage. Furthermore, t (s) and i (A) refer to the discharge time and charge/discharge current, respectively.

**Theoretical analysis.**

We used the DFT as implemented in the Vienna Ab initio simulation package (VASP) in all calculations. The exchange-correlation potential is described by using the generalized gradient approximation of Perdew-Burke-Ernzerhof (GGA-PBE). The projector augmented-wave (PAW) method is employed to treat interactions between ion cores and valence electrons. The plane-wave cutoff energy was fixed to 400 eV. Given structural models were relaxed until the Hellmann-Feynman forces smaller than -0.02 eV/Å and the change in energy smaller than 10^-5^ eV was attained. The long-range van der Waals interaction is described by the DFT-D3 approach.

The adsorption energy (Eads) and binding energy of species are calculated by:

E_ads_ = E_(system)_-E_(catalyst)_ -E_(species)_

E_bin_ = (E_(species)_+ E_(catalyst)_)- E_(system)_

where E_(system)_, E_(catalyst)_, and E_(species)_ are the total energy of the optimized system with adsorbed species, the isolated catalyst, and species, respectively.


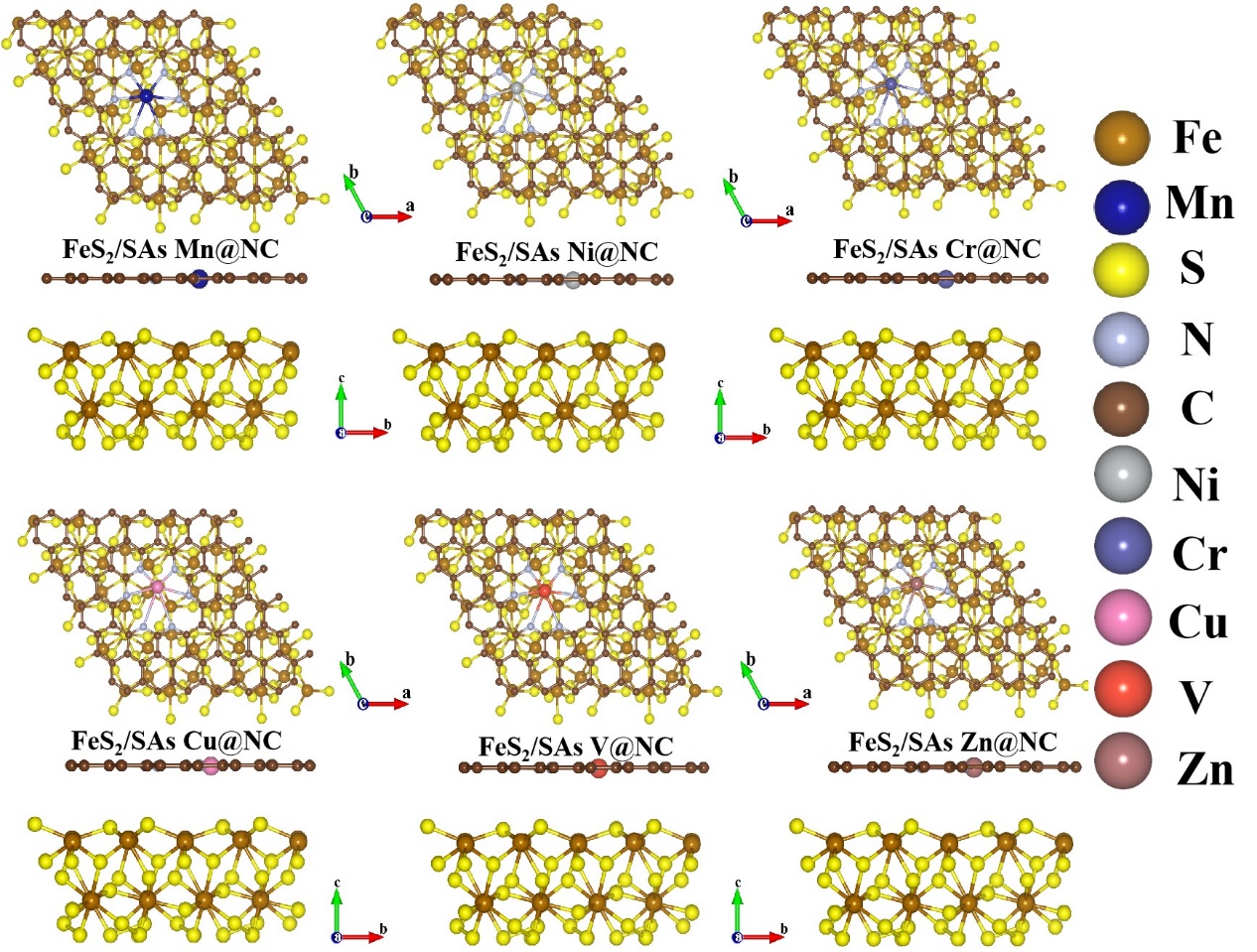


**Figure S1****.** Adsorption configurations of FeS_2_ on SAs Mn@NC, SAs Ni@NC, SAs Cr@NC, SAs Cu@NC, SAs V@NC, and SAs Zn@NC.


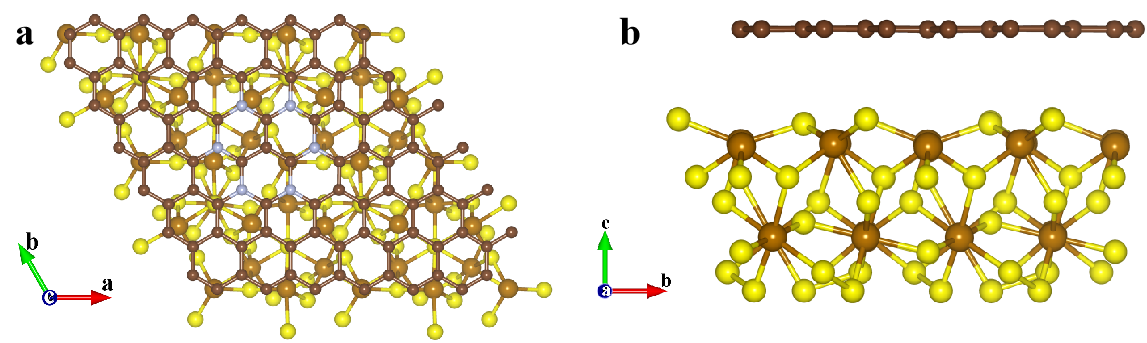


**Figure S2.** Adsorption configurations of FeS_2_ on NC: (a) top view, (b) side view.


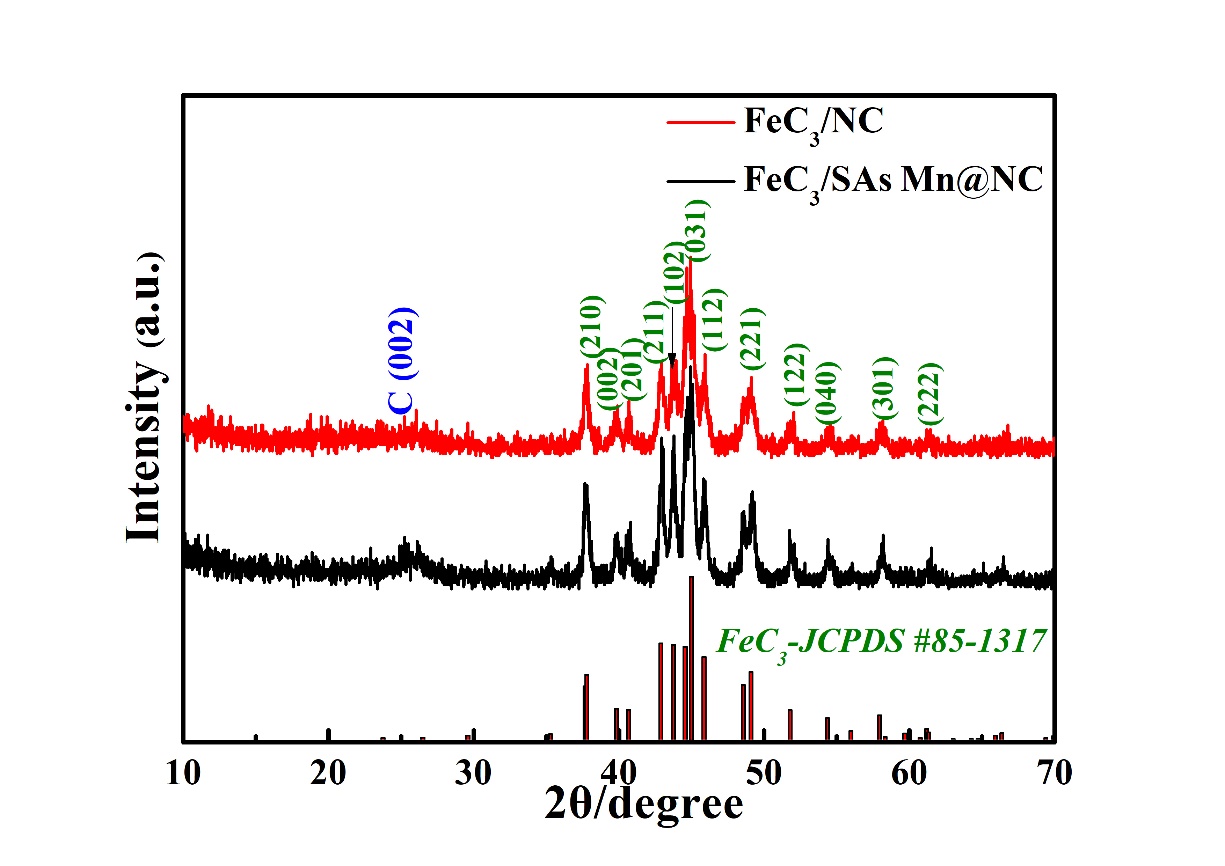


**Figure S3.** XRD patterns for FeC_3_/SAs Mn@NC and FeC_3_/NC.


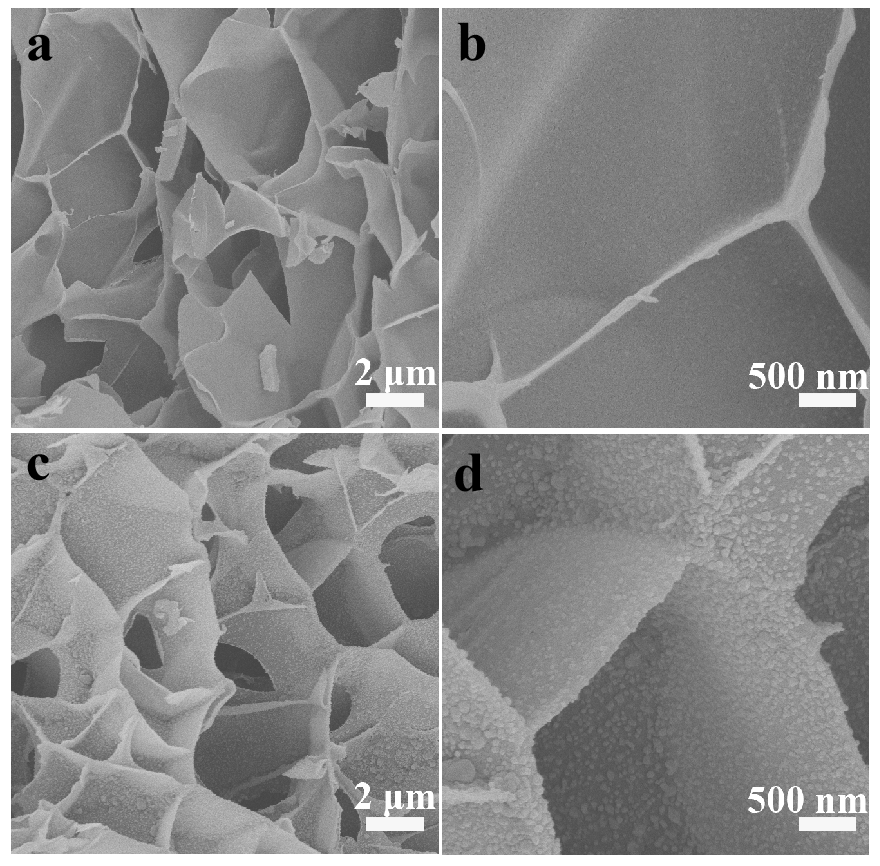


**Figure S4.** FESEM images for (a, b) FeC_3_/NC and (c, d) FeS_2_/NC.


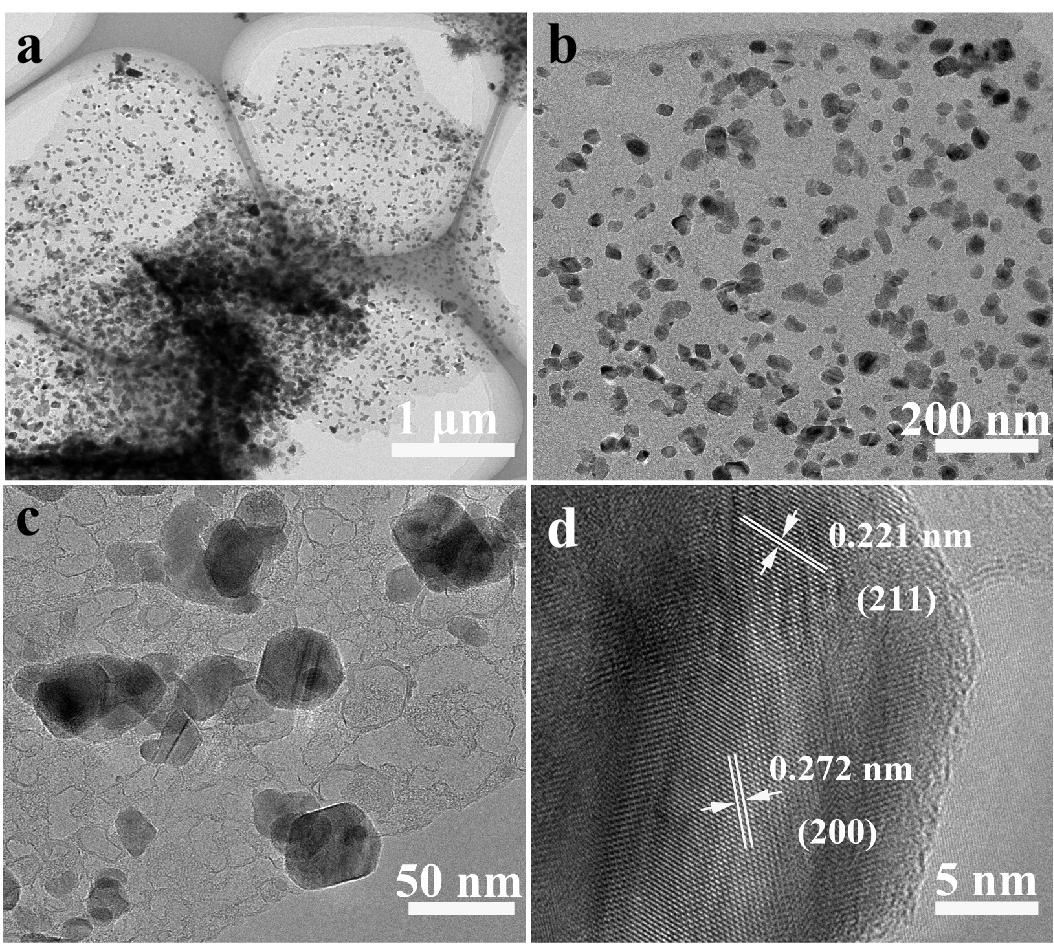


**Figure S5.** (a-c) TEM and (d) HRTEM images of FeS_2_/NC.


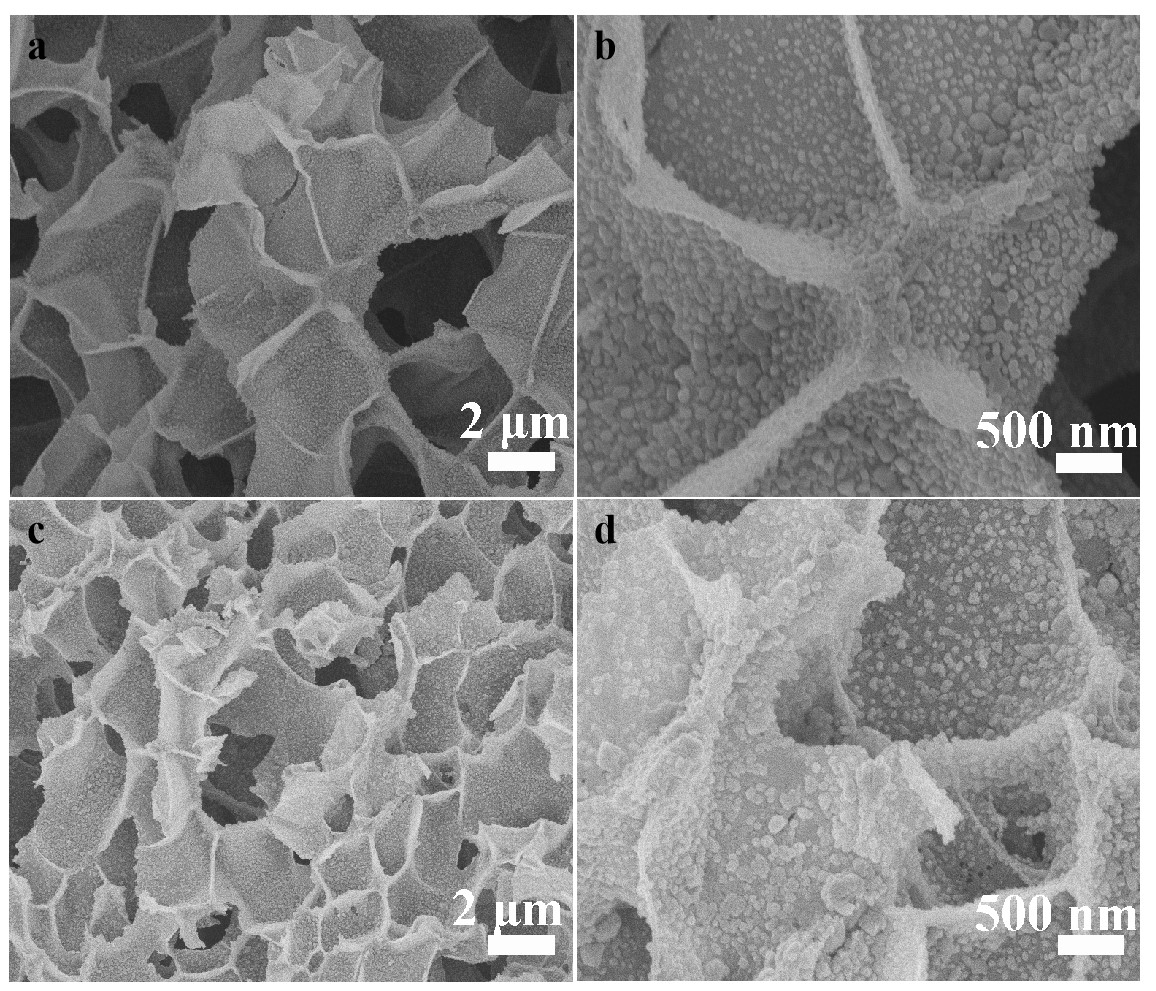


**Figure S6.** FESEM images for (a, b) FeS_2_/SAs [Mn@NC-0.025](mailto:Mn@NC-0.025) and (c, d) FeS_2_/SAs [Mn@NC-0.05](mailto:Mn@NC-0.025).


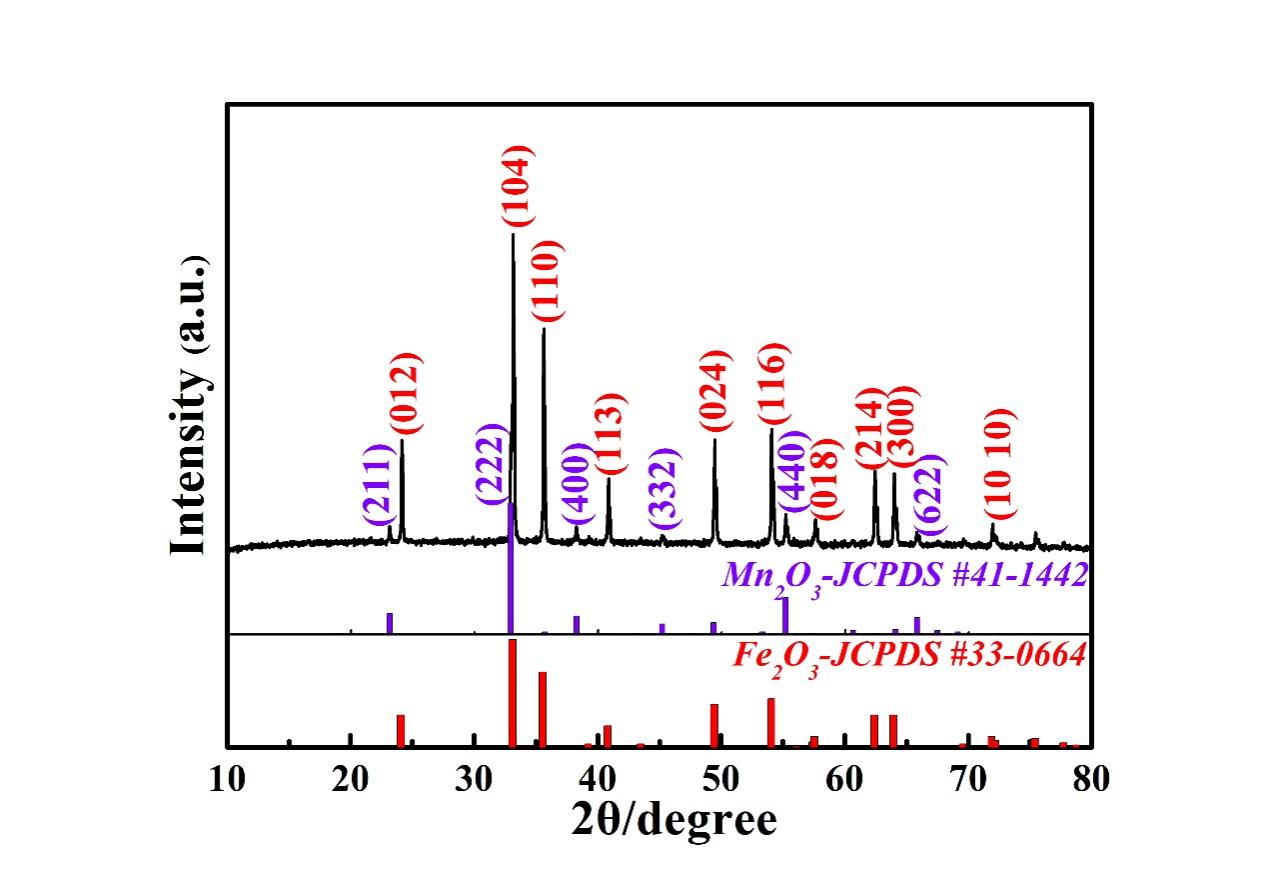


**Figure S7.** XRD pattern of combustion products of FeS_2_/SAs Mn@NC.


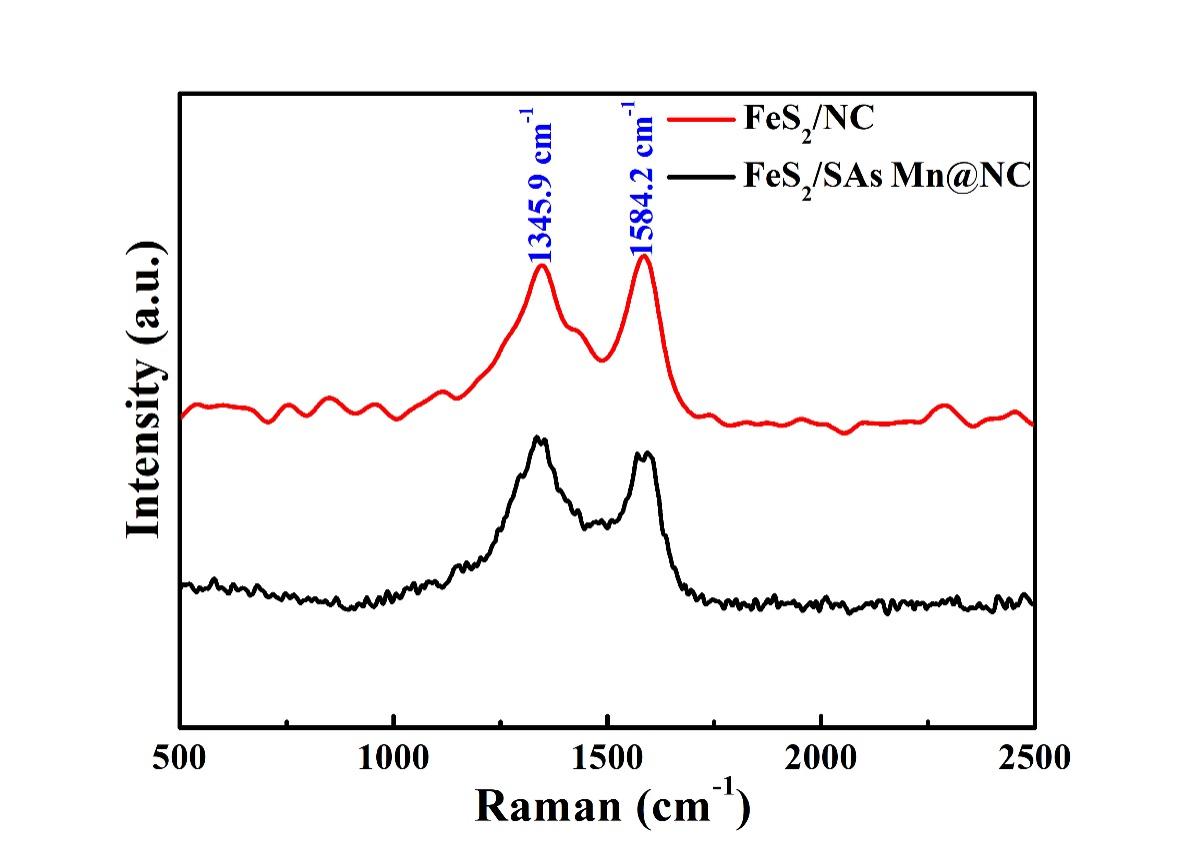


**Figure S8.** Raman spectra of FeS_2_/SAs Mn@NC and FeS_2_/NC.


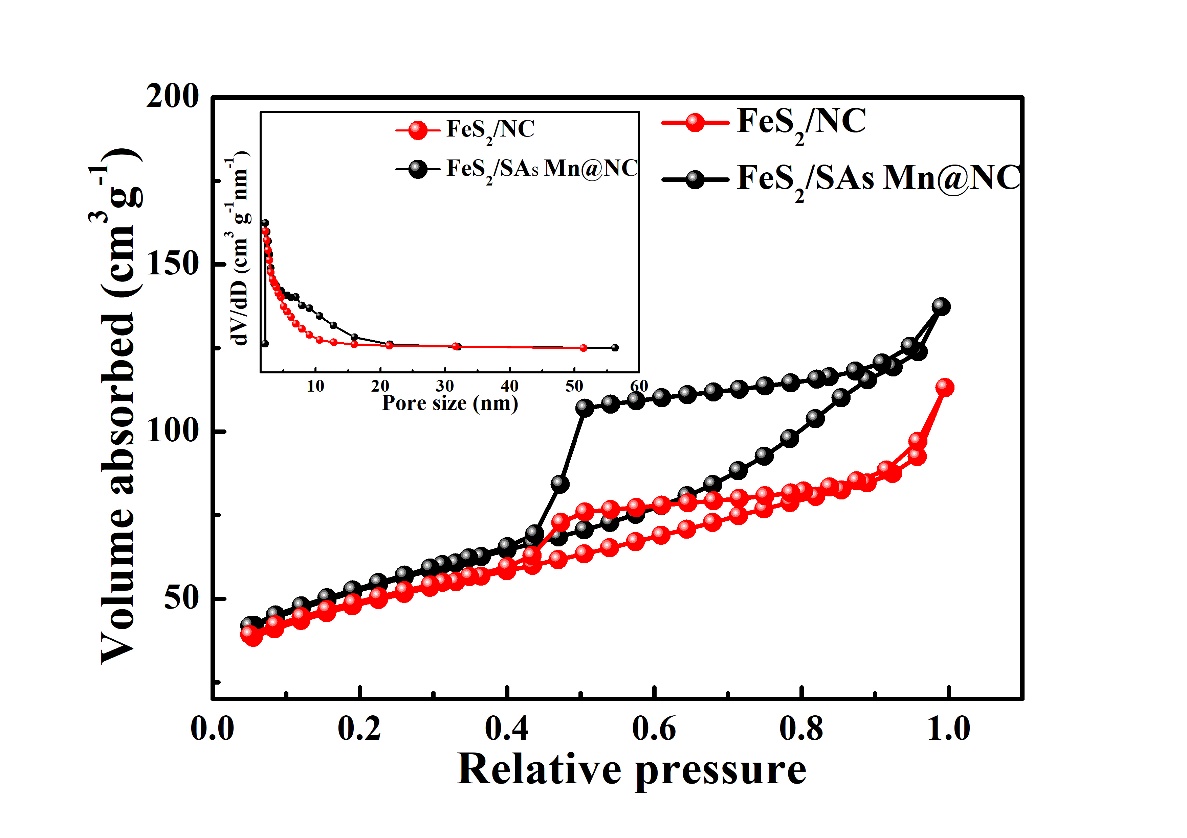


**Figure S9.** Nitrogen adsorption-desorption isothermal curves of FeS_2_/SAs Mn@NC and FeS_2_/NC (inset: pore distribution curves of FeS_2_/SAs Mn@NC and FeS_2_/NC).


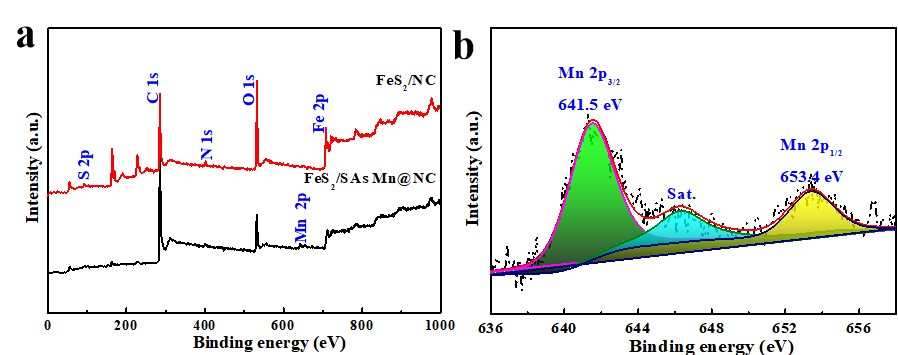


**Figure S10.** (a) XPS survey spectra of FeS_2_/SAs Mn@NC and FeS_2_/NC. (b) High-resolution Mn 2p spectrum of FeS_2_/SAs Mn@NC.


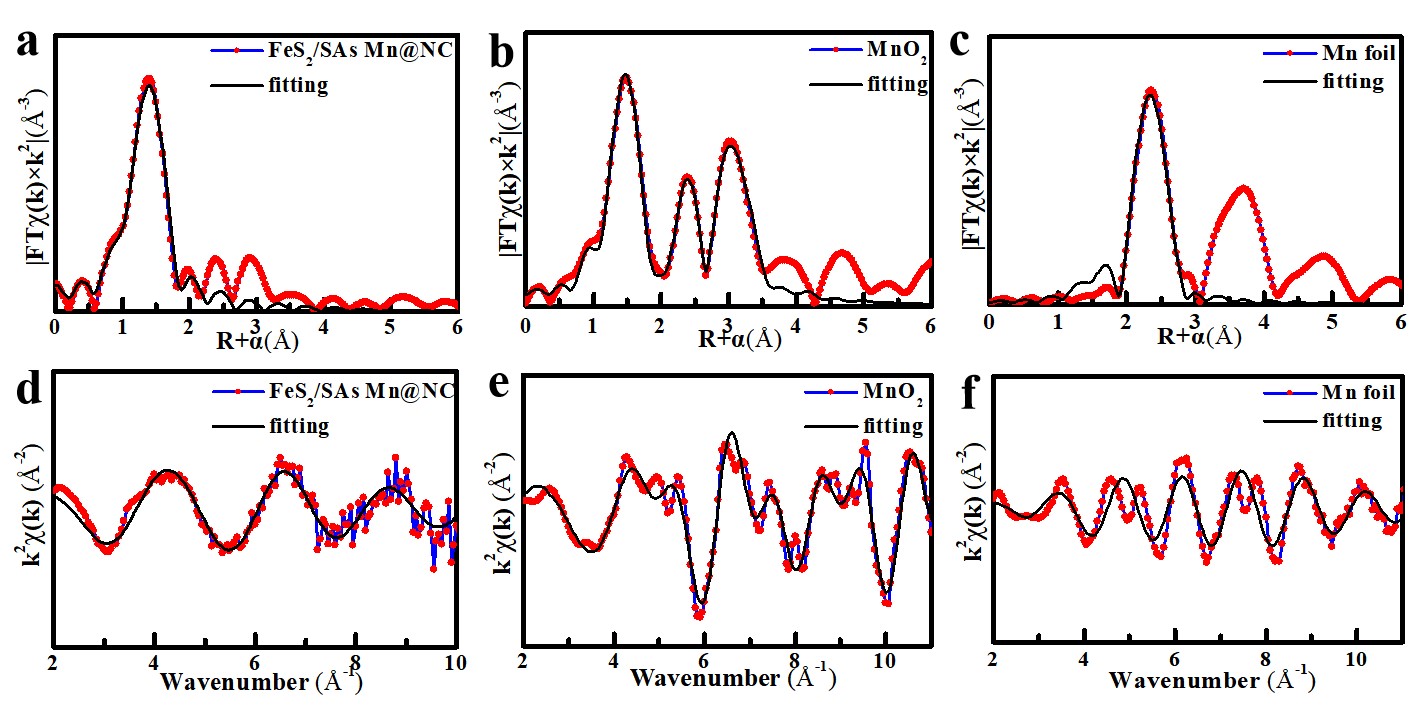


**Figure S11.** Fitted EXAFS data of Mn K-edge in K space for (a, d) FeS_2_/SAs Mn@NC, (b, e) MnO_2_, and (c, f) commercial Mn foil.


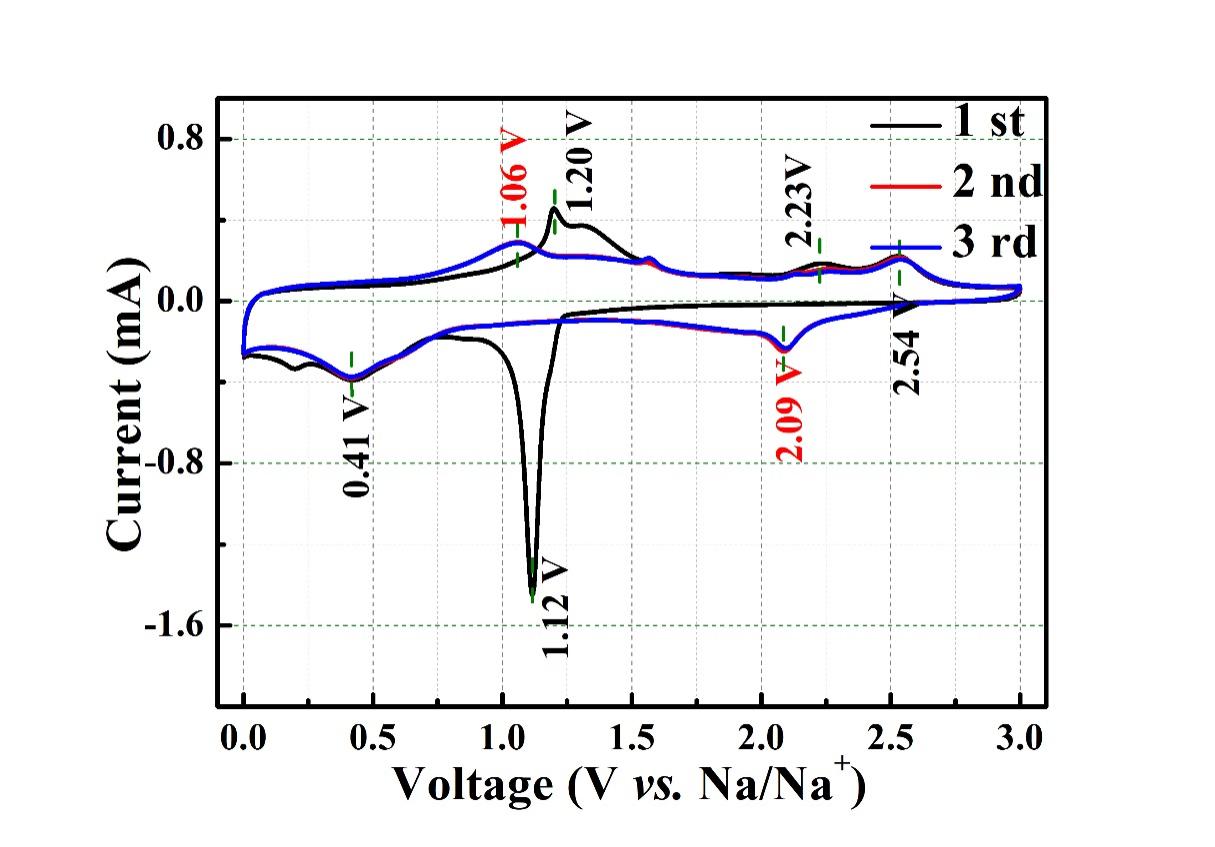


**Figure S12.** Initial three CV curves of FeS_2_/NC at 0.5 mV s^-1^.


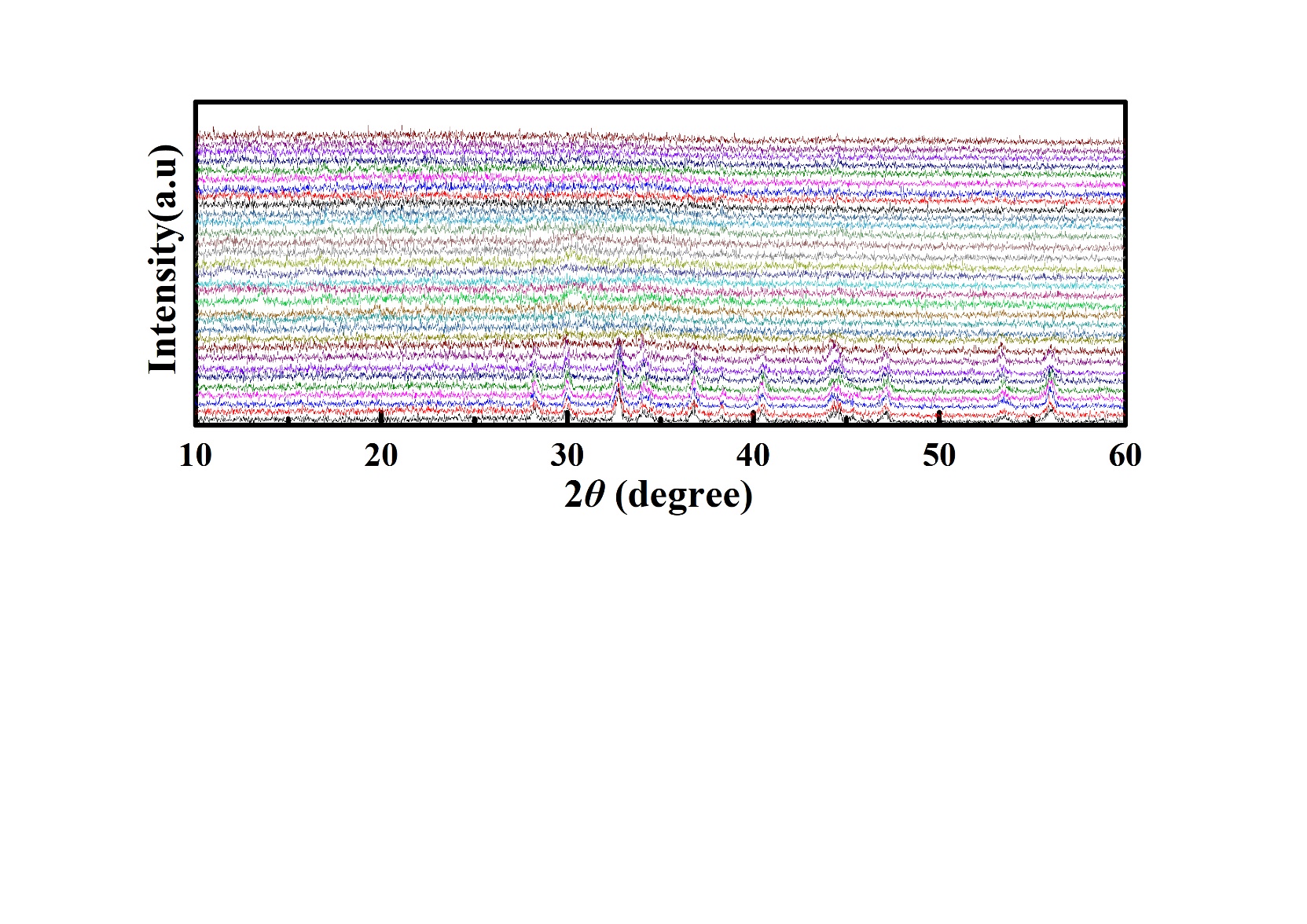


**Figure S13.** Ex-situ XRD patterns of FeS_2_/SAs Mn@NC at different discharge-charge states.


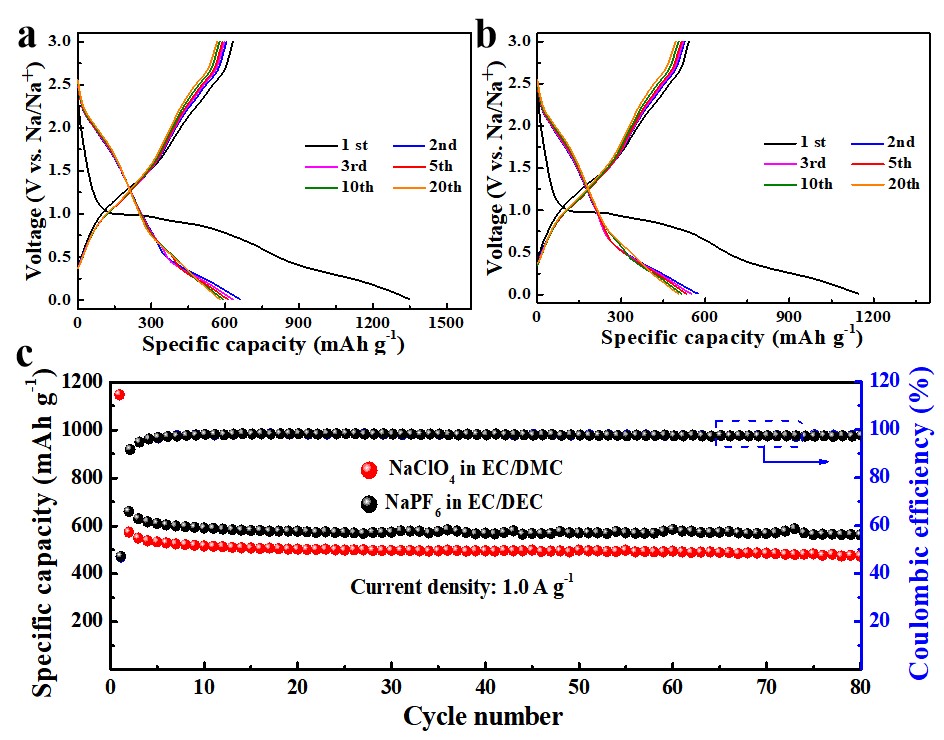


**Figure S14.** GCD profiles of FeS_2_/SAs Mn@NC at different electrolytes: (a) 1 M NaPF_6_ in EC/DEC, (b) 1 M NaClO_4_ in EC/DME. (c) Comparison of cycling stability of FeS_2_/SAs Mn@NC at different electrolytes.

**Description:** To further reveal the effects of electrolyte on electrochemical performance of FeS_2_/SAs Mn@NC, we performed additional testing with different ester-based electrolytes (1 M NaClO_4_ in EC/DMC and 1 M NaPF_6_ in EC/DEC) to confirm if the 1M NaCF_3_SO_3_ in DEGDME electrolyte yields the best cycle life; the results are compared in Figure S14. Clearly, FeS_2_/SAs Mn@NC shows the almost same GCD curves in two ester electrolytes (Figure S14a, b). Meanwhile, as is evident that FeS_2_/SAs Mn@NC delivers the initial discharge/charge capacity of 1348.9/630.9 mAh g^-1^ (1 M NaPF_6_ in EC/DEC,) and 1146.2/540.8 mAh g^-1^ (1 M NaClO_4_ in EC/DME) at 1.0 A g^-1^, respectively, with the lower ICE values of only 46.8% and 47.2%. After 80 cycles, the capacities using 1 M NaClO_4_ in EC/DMC and 1 M NaPF_6_ in EC/DEC can be maintained to be about 549.3 and 461.6 mAh g^−1^ (Figure S14). In contrast, the battery with 1 M NaCF_3_SO_3_ in DEGDME electrolyte shows a much stable cycle life, maintaining a reversible capacity of 658.6 mAh g^-1^ after 80 cycles with a high coulombic efficiency of 99% (see Figure 5a), further confirming that the ether-based electrolyte can significantly enhance the electrochemical performance of FeS_2_/SAs Mn@NC.


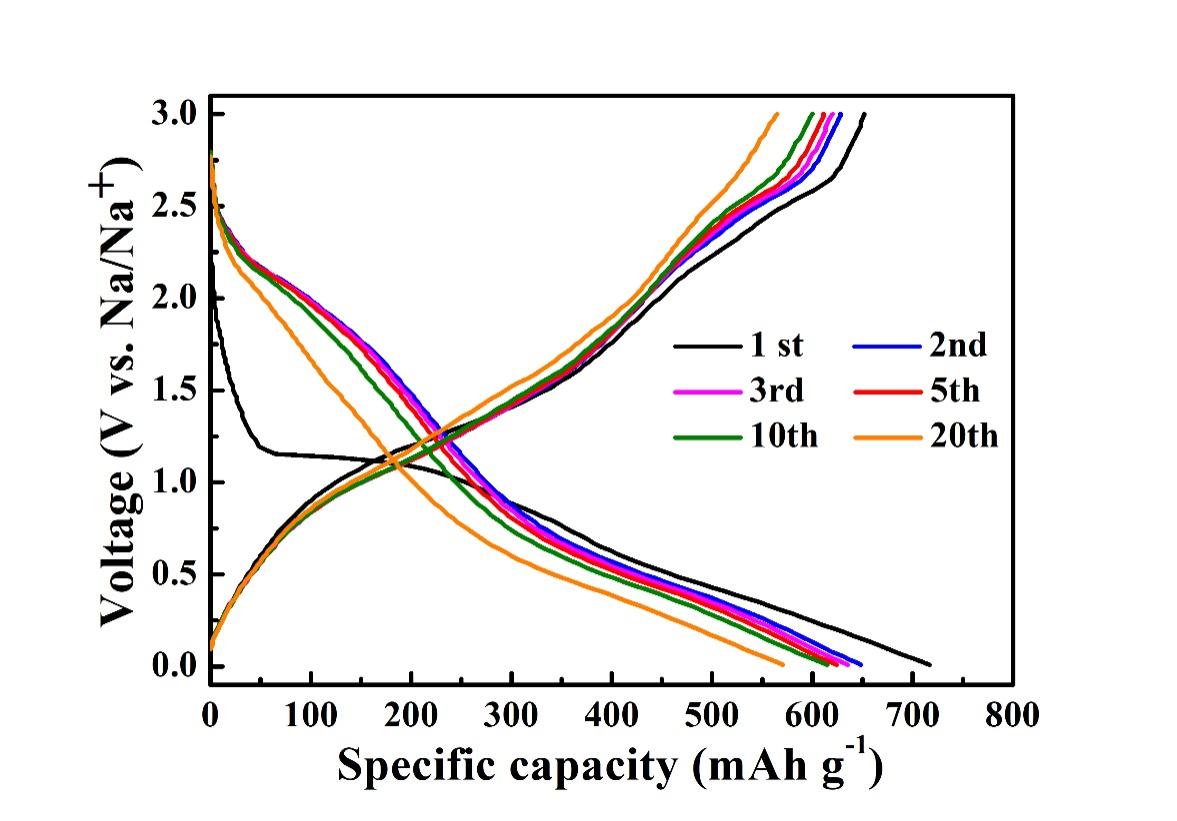


**Figure S15.** GCD profiles of FeS_2_/NC at 1.0 A g^-1^.


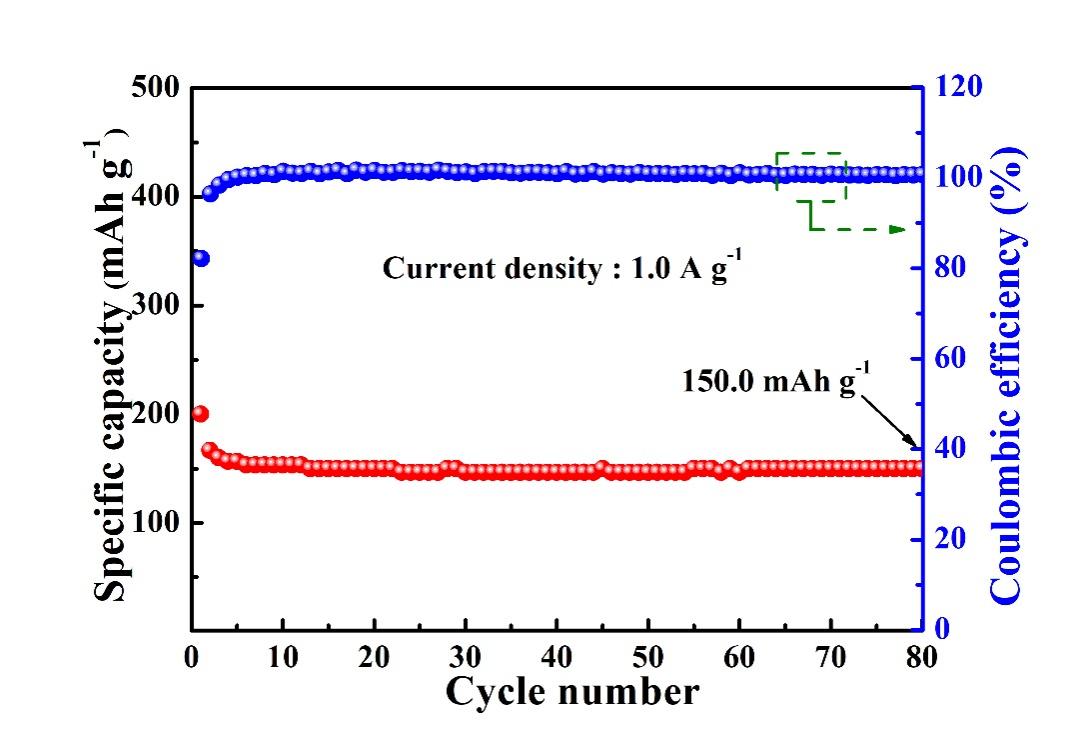


**Figure S1****6.** Cycling performance of NC at 1.0 A g^-1^.


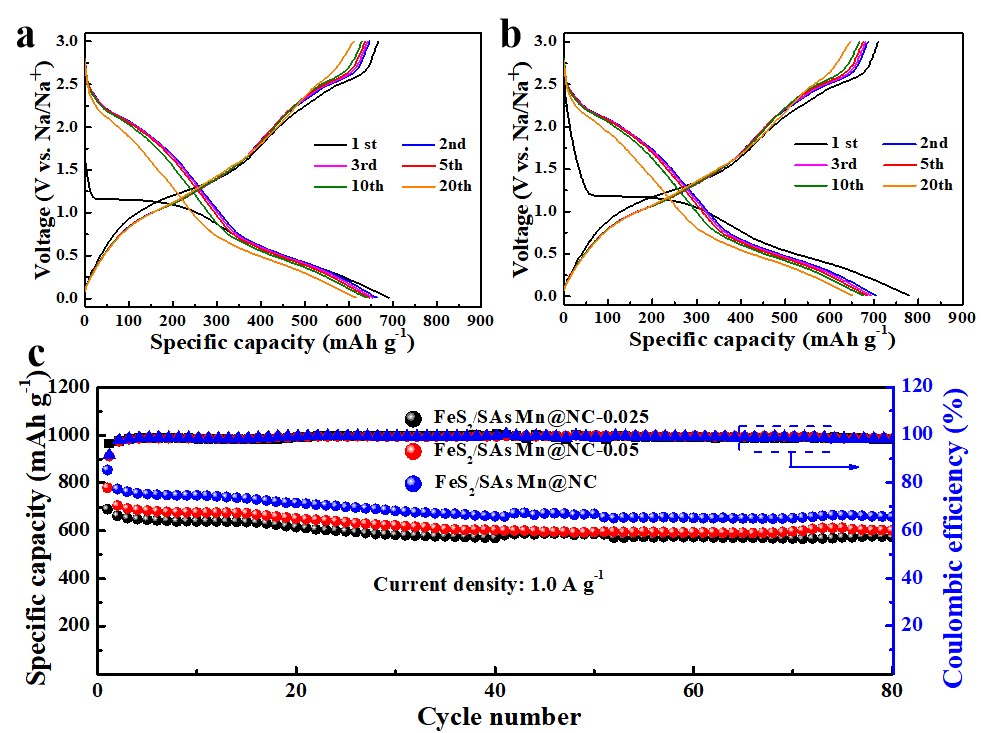


**Figure S17.** GCD profiles of (a) FeS_2_/SAs [Mn@NC-0.025](mailto:Mn@NC-0.025) and (b) FeS_2_/SAs [Mn@NC-0.05](mailto:Mn@NC-0.025) at 1.0 A g^-1^. (c) Comparison of cycling performance of FeS_2_/SAs [Mn@NC-0.025](mailto:Mn@NC-0.025), FeS_2_/SAs [Mn@NC-0.05](mailto:Mn@NC-0.025) and FeS_2_/SAs [Mn@NC](mailto:Mn@NC-0.025) at 1.0 A g^-1^.


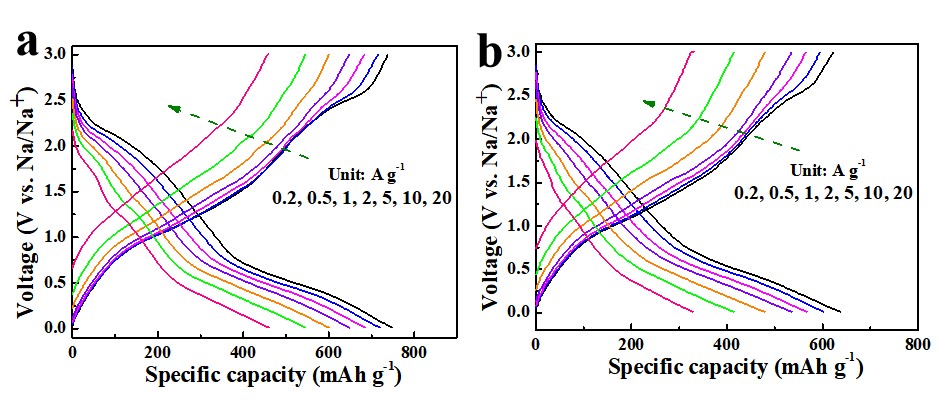


**Figure S18.** GCD profiles of (a) FeS_2_/SAs Mn@NC and (b) FeS_2_/NC at different rates.


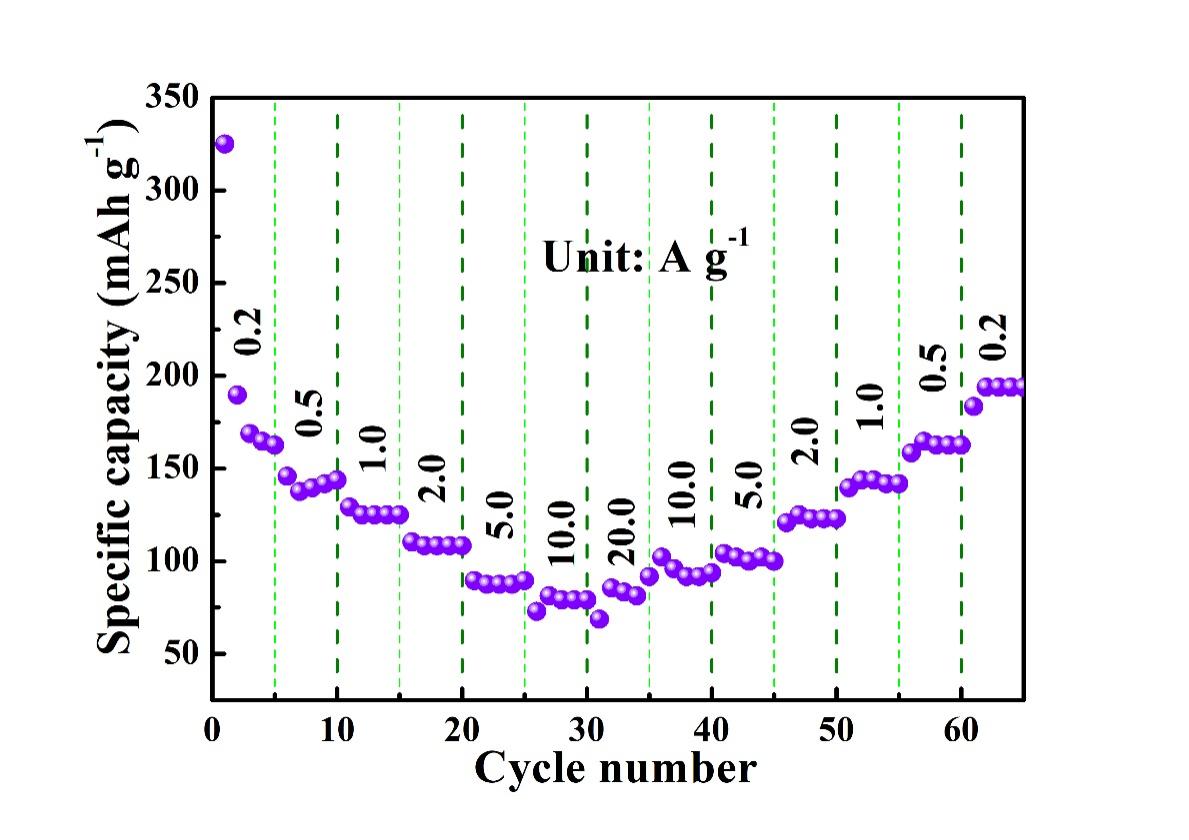


**Figure S19.** Rate performance of NC at different rates.


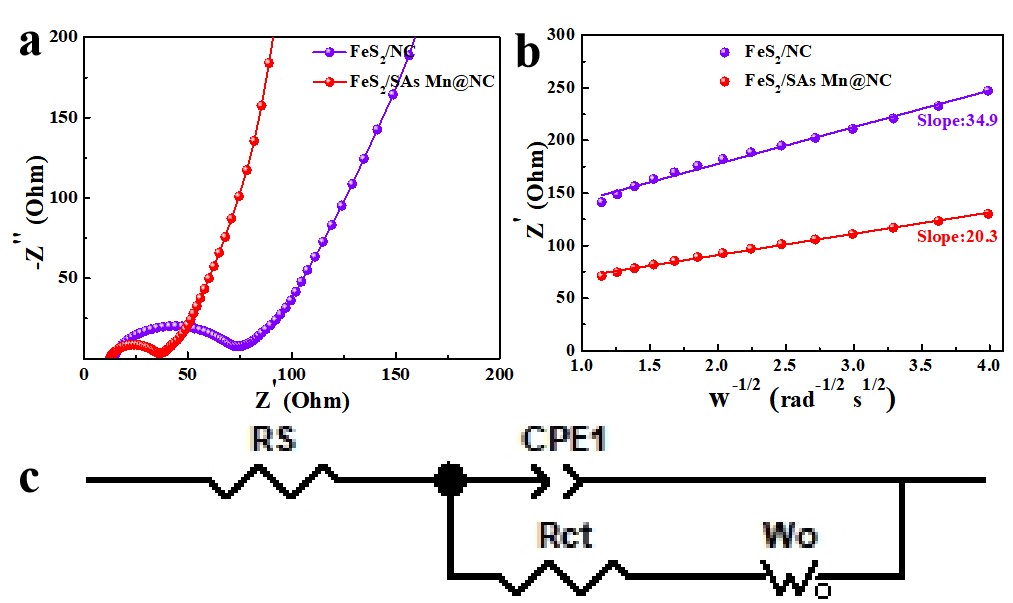


**Figure S20.** (a) EIS spectra (b) the plots of ω^1/2^ versus Z’ curves in the low frequency region for FeS_2_/SAs Mn@NC and FeS_2_/NC. (c) The equivalent circuits of EIS spectra.


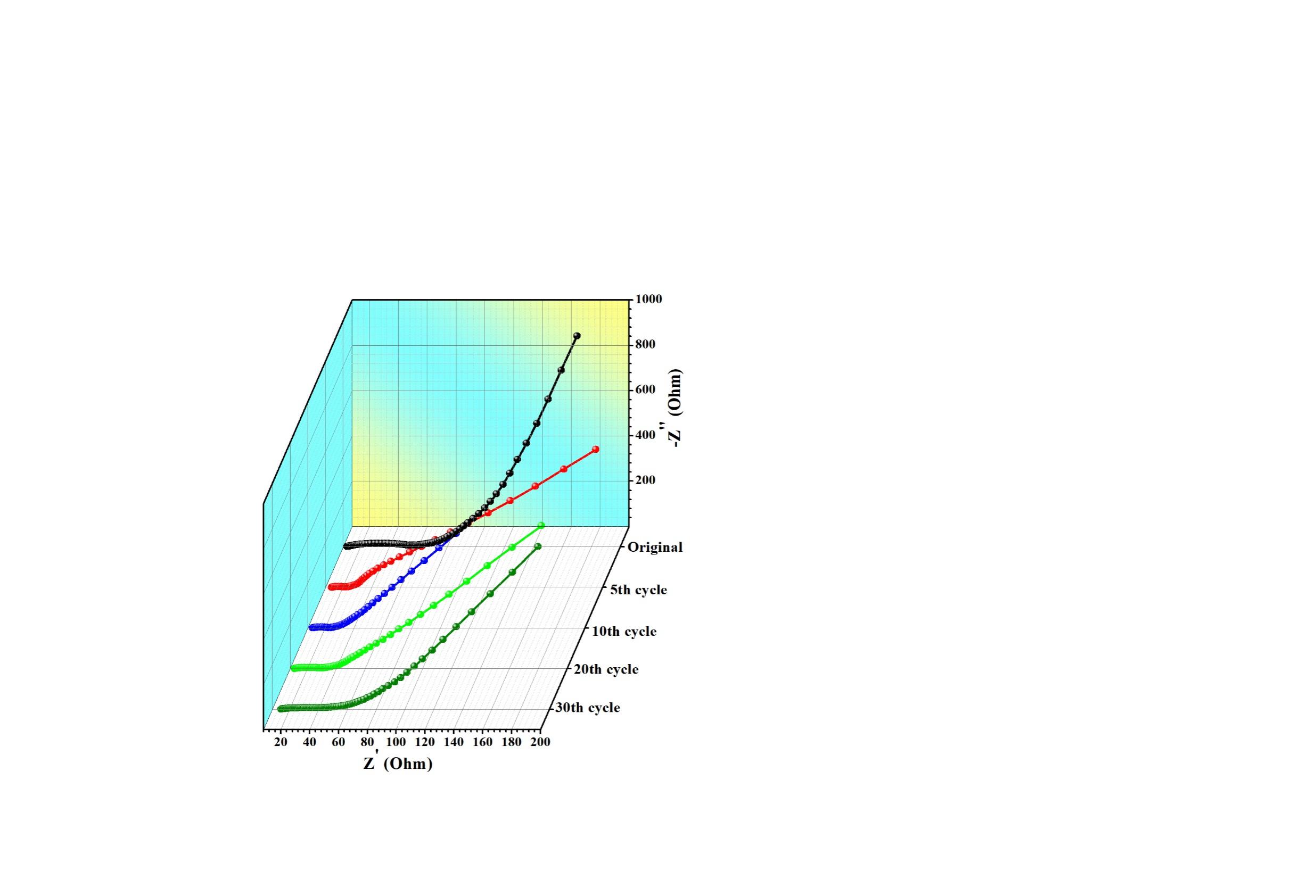


**Figure S21.** EIS spectra of FeS_2_/SAs Mn@NC at different cycles.


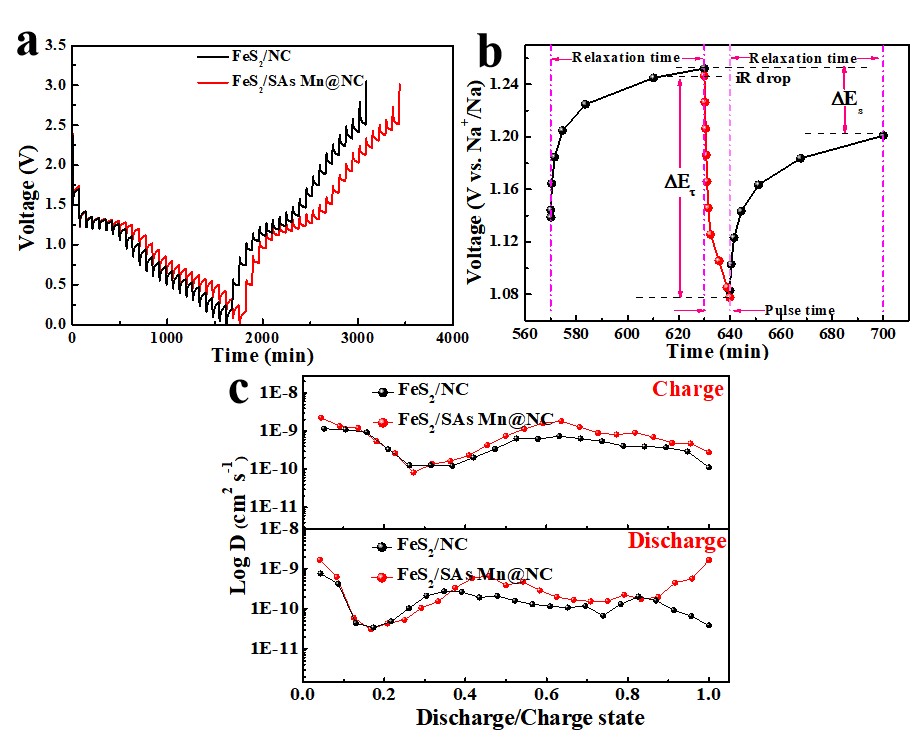


**Figure S22.** (a) GITT curves of FeS_2_/SAs Mn@NC and FeS_2_/NC. (b) voltage response with time for FeS_2_/SAs Mn@NC at a single current pulse. (c) Na^+^ diffusion coefficients for FeS_2_/SAs Mn@NC and FeS_2_/NC during the discharge and charge process.

**GITT description:** The diffusion coefficient of Na^+^ ion (D_Na+_) in three electrodes is calculated from the galvanostatic intermittent titration technique (GITT) potential profiles. During the GITT measurements, the cell was charged and discharged at 0.1 A·g^-1^ for 10 min followed by an open-circuit stand for 60 min until a steady state was achieved to the voltage range 0.01–3.0 V. The sodium diffusion coefficient (DNa+) can be figured out using the simplified Fick’s second law:

$$D=\frac{4L^{2}}{\pi\tau}\left(\frac{\Delta E_{S}}{\Delta E_{\tau}}\right)$$

Among them, τ (s) represents the constant current pulse time. L (cm) refers to the sodium ion diffusion distance, which can be identified as the thickness of the electrodes for compact electrode. ΔE_s_ (V) is the voltage change caused by the pulse, and ΔE_τ_ is the voltage change during the constant current charging and discharging process.


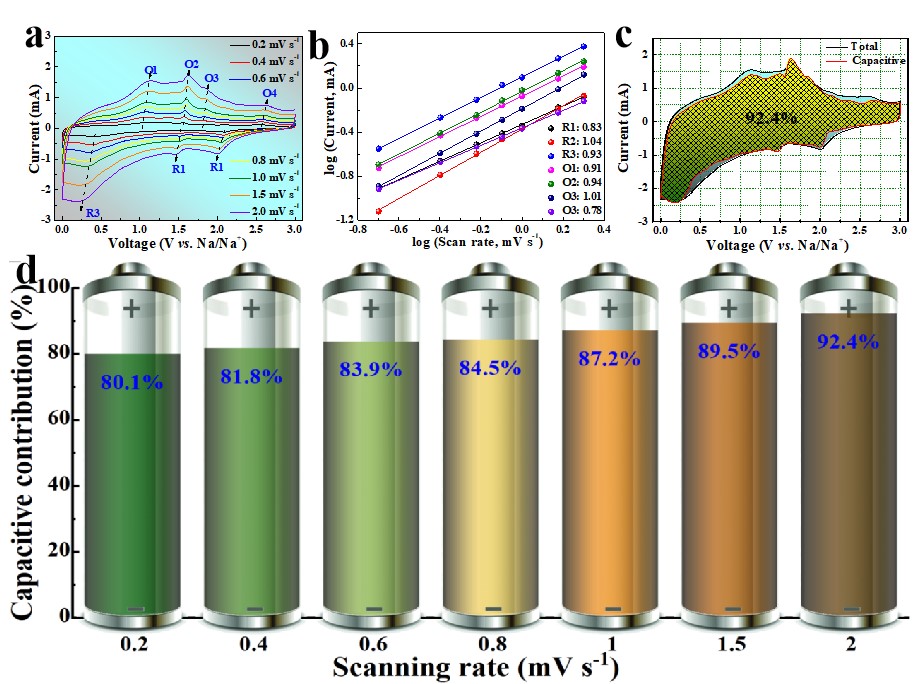


**Figure S****23.** (a) CV curves of FeS_2_/NC at diverse scan rates. (b) A series of b-values according to the plot of log *i* versus log *v* of redox peaks from CV scans. (c) Diagrammatic drawing of capacitive contribution for FeS_2_/NC at 2.0 mV s^-1^. (d) Normalized contribution percentage of capacitively controlled charges from 0.2 to 2.0 mV s^-1^.


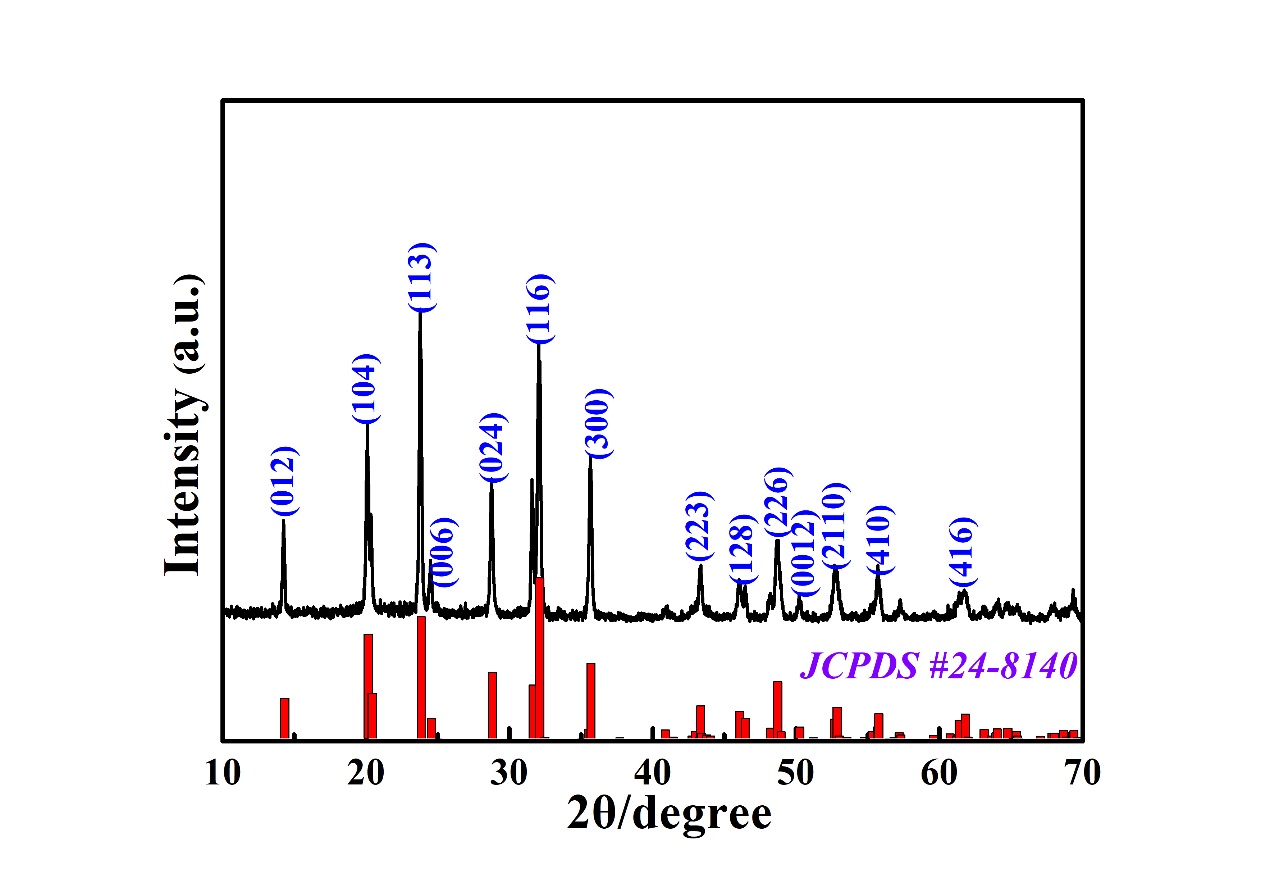


**Figure S24.** XRD pattern of NVP@C.


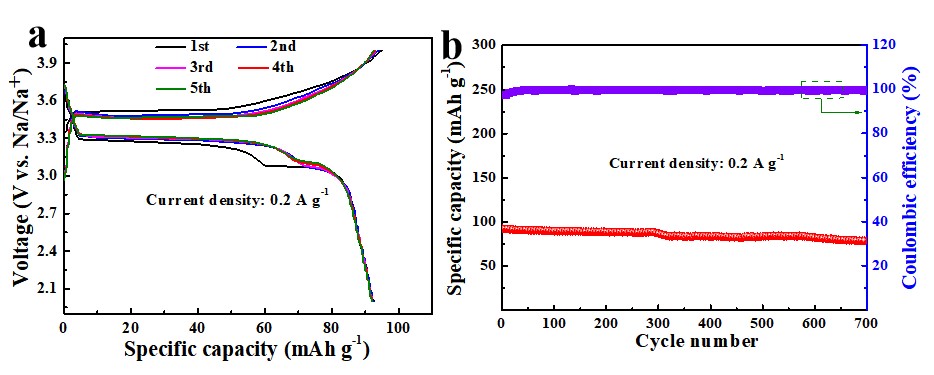


**Figure S2****5.** (a) Initial five GCD profiles and (b) cycling performance of NVP@C cathode at 0.2 A g^-1^.


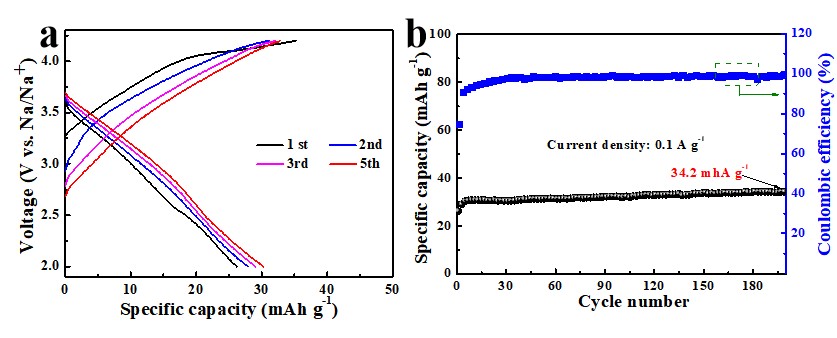


**Figure S26.** (a) GCD profiles and (b) cycling stability of AC at 0.1 A g^-1^.


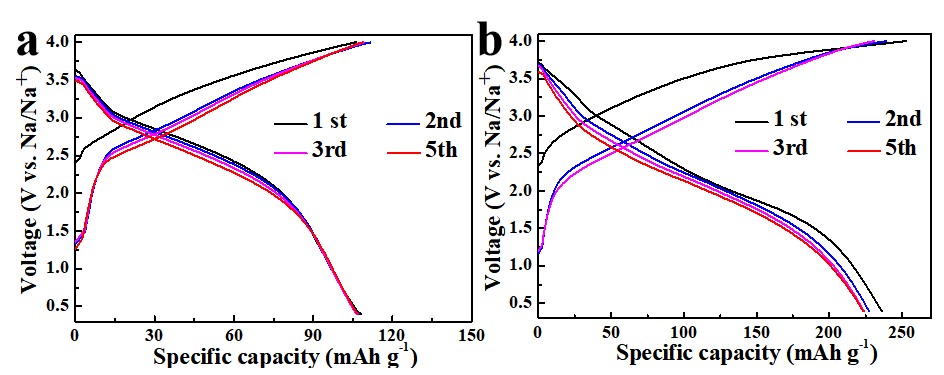


**Figure S27.** Initial five GCD profiles of FeS_2_/SAs Mn@NC//NVP@C SIFCs with different mass ratios of A:C at 1.0 A g^-1^: (a) A:C~1:2, (b) A:C~1:4.


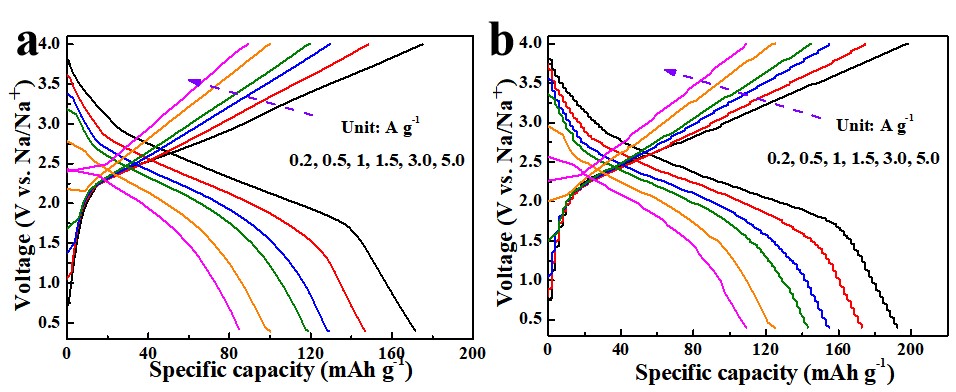


**Figure S28.** GCD profiles of FeS_2_/SAs Mn@NC//NVP@C SIFCs with different mass ratios of A:C at different rates: (a) A:C~1:2, (b) A:C~1:4.


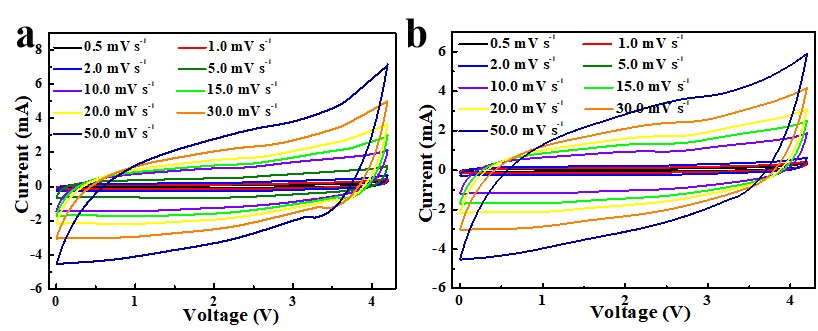


**Figure S2****9.** CV curves of FeS_2_/SAs Mn@NC//AC SIHCs with the different mass ratios of A:C in the scan range of 0.5 to 50.0 mV s^-1^: (a) A:C~1:4, (b) A:C~1:6.


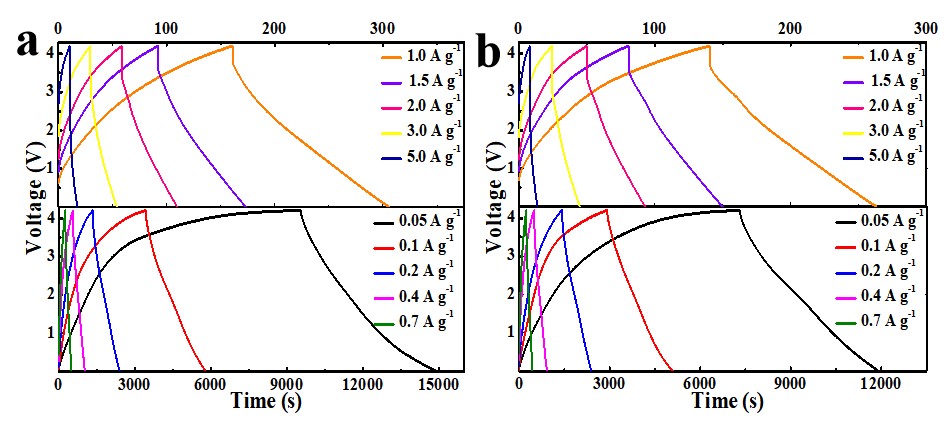


**Figure S****30.** GCD profiles of FeS_2_/SAs Mn@NC//AC SIHCs with the different mass ratios of A:C : (a) A:C~1:4, (b) A:C~1:6.

**Table S1.** EXAFS fitting parameters at the Mn *K*-edge for various samples.

| Sample | Shell | *CN^a^* | *R*(Å)*^b^* | *σ*^2^(Å^2^)*^c^* | Δ*E*_0_(eV)*^d^* | *R* factor |
| --- | --- | --- | --- | --- | --- | --- |
| Mn-foil | Mn-Mn | 2* | 3.349±0.001 | 0.0192±0.0033 | -5.3±0.1 | 0.0104 |
|  | Mn-Mn | 4* | 2.672±0.001 | 0.0115±0.0001 | 6.0±0.6 |  |
| MnO_2_ | Mn-O | 6.0±0.4 | 1.881±0.001 | 0.0021±0.0008 | 0.7±0.5 | 0.0039 |
|  | Mn-Mn | 4.4±0.5 | 2.852±0.001 | 0.0083±0.0001 | -8.0±1.0 |  |
|  | Mn-Mn | 5.9±1.0 | 3.450±0.001 | 0.0025±0.0013 | 1.2±0.6 |  |
| sample-Mn | Mn-C/N | 5.6±0.9 | 1.937±0.001 | 0.0076±0.0024 | -9.0±0.9 | 0.0068 |

*^a^CN*, coordination number; *^b^R*, the distance to the neighboring atom; *^c^σ*^2^, Debye-Waller factor, the Mean Square Relative Displacement (MSRD); *^d^ΔE*_0_, inner potential correction; *R* factor indicates the goodness of the fit. *S*0^2^ was fixed to 0.835, according to the experimental EXAFS fit of Mn foil by fixing *CN* as the known crystallographic value. * This value was fixed during EXAFS fitting, based on the known structure of Mn. Fitting range: 3.0 ≤ *k* (/Å) ≤ 11.0 and 1.8 ≤ *R* (Å) ≤ 2.8 (Mn foil); 3.0 ≤ *k* (/Å) ≤ 11.0 and 1.0 ≤ *R* (Å) ≤ 3.5 (MnO2); 2.5 ≤ *k* (/Å) ≤ 10.0 and 1.0 ≤ *R* (Å) ≤ 2.0 (sample-Mn). A reasonable range of EXAFS fitting parameters: 0.700 < *Ѕ*_0_^2^ < 1.000; *CN >* 0; *σ*^2^ > 0 Å^2^; |Δ*E*_0_| < 15 eV; *R* factor < 0.02.

**Table S2.** Comparison of cycle lifespan and specific capacity of FeS_2_/SAs Mn@NC with previously reported FeS_2_-based anodes in SIBs systems.

| **Materials** | **Voltage window (V)** | **Rate**  **(A g ^-1^)** | **Cycle number** | **Capacity**  **(mAh g^-1^)** | **Electrolyte** | **Ref.** |
| --- | --- | --- | --- | --- | --- | --- |
| FeS_2_@NSC/G | 0.01-2.5 | 10.0 | 400 | 203.5 | 1.0 M NaCF_3_SO_3_ in DIGLYME | 1 |
| Co doped FeS_2_ | 0.8-2.9 | 20.0 | 500 | 172.0 | 1.0 M NaCF_3_SO_3_ in DIGLYME | 2 |
| FeS_2_@CF-NS | 0.01-3.0 | 5.0 | 700 | 431.1 | 1 M NaPF_6_ in DIGDME | 3 |
| FeS_2_-C/RG | 0.01-3.0 | 6.0 | 1000 | 291.7 | 1 M NaClO_4_ in EC/DEC | 4 |
| CuS/FeS_2_ | 0.01-3.0 | 5.0 | 300 | 537.0 | 1 M NaPF_6_ in DEGDME | 5 |
| MoS_2_/FeS_2_/C | 0.01-2.8 | 5.0 | 1400 | 414.3 | 1 M NaClO_4_ in EC/DEC | 6 |
| CHS-FeS_2_ | 0.01-3.0 | 1.0 | 400 | 305.5 | 1.0 M NaClO_4_ in EC: DMC: DMC | 7 |
| Fe_7_S_8_/FeS_2_/NCNT | 0.01-3.0 | 1.0 | 1000 | 403.2 | 1 M NaPF_6_ in DEGDME | 8 |
| FeS_2_@C/HRGO | 0.01-3.0 | 5.0 | 1200 | 252.0 | 1 M NaClO_4_ in EC/DMC | 9 |
| Hollow FeS_2_(M) | 0.8-2.8 | 0.1 | 300 | 363.0 | 1.0 M NaCF_3_SO_3_ in DEGDME | 10 |
| FeS_2_/Mn SAs@NC | **0.01-3.0** | **20.0** | **2250** | **405.9** | 1.0 M NaCF_3_SO_3_ in DEGDME | **This work** |

**Table S3.** Fitting result of EIS spectra in Fig. S20 with the equivalent circuit proposed.

| **Cycle number** | **R_s_ (Ω)** | **R_ct_ (Ω)** |
| --- | --- | --- |
| **FeS_2_/SAs Mn@NC** | 12.4 | 24.8 |
| **FeS_2_/NC** | 13.8 | 60.9 |

**References**

1. M. Shao, Y. Cheng, T. Zhan, S. Li, W. Zhang, B. Zheng, J. Wu, W. Xiong, F. Huo, J. Lu, *ACS Appl. Mater. Interfaces* **2018**, *10*, 33097–33104.
2. [K. Zhang](https://onlinelibrary.wiley.com/authored-by/Zhang/Kai), [M. Park](https://onlinelibrary.wiley.com/authored-by/Park/Mihui), [L. Zhou](https://onlinelibrary.wiley.com/authored-by/Zhou/Limin), [G. Lee](https://onlinelibrary.wiley.com/authored-by/Lee/Gi%E2%80%90Hyeok), [J. Shin](https://onlinelibrary.wiley.com/authored-by/Shin/Jeongyim), [Z. Hu](https://onlinelibrary.wiley.com/authored-by/Hu/Zhe), [S. Chou](https://onlinelibrary.wiley.com/authored-by/Chou/Shu%E2%80%90Lei), [J. Chen](https://onlinelibrary.wiley.com/authored-by/Chen/Jun), [Y. Kang](https://onlinelibrary.wiley.com/authored-by/Kang/Yong%E2%80%90Mook), *Angew. Chem., Int. Ed*. **2016**, *55*, 12822–12826.
3. Z. Lu, Y. Zhai, N. Wang, Y. Zhang, P. Xue, M. Guo, B. Tang, D. Huang, W. Wang, Z. Bai, S. Dou, *Chem. Eng. J.* **2020**, *380*, 122455.
4. F. Wang, W. Zhang, [H. Zhou](https://www.sciencedirect.com/author/7404742194/haihui-zhou), H. Chen, Z. Huang, Z. Yan, R. Jiang, C. Wang, Z. Tan, Y. Kuang, *[Chem. Eng. J.](https://www.sciencedirect.com/journal/chemical-engineering-journal)* **2020**, *[380](https://www.sciencedirect.com/journal/chemical-engineering-journal/vol/380/suppl/C)*, 122549.
5. J. Je, H. Lim, H. W. Jung, S. Kim, *Small* **2022**, *18*, 2105310.
6. L. Ma, X. Zhou, J. Sun, P. Zhang, B. Hou, S. Zhang, N. Shang, J. Song, H. Ye, H. Shao, Y. Tang, X. Zhao, *[J. Energy Chem.](https://www.sciencedirect.com/journal/journal-of-energy-chemistry)* **2023**, *[82](https://www.sciencedirect.com/journal/journal-of-energy-chemistry/vol/82/suppl/C)*, 268–276.
7. L. Ma, B. Hou, H.i Zhang, S. Yuan, B. Zhao, Y. Liu, X. Qi, [H. Liu](https://www.sciencedirect.com/author/56335747500/haiyan-liu), S. Zhang, J. Song, X. Zhao, *[Chem. Eng. J.](https://www.sciencedirect.com/journal/chemical-engineering-journal)* **2023**, *[453](https://www.sciencedirect.com/journal/chemical-engineering-journal/vol/453/part/P2)*[,](https://www.sciencedirect.com/journal/chemical-engineering-journal/vol/453/part/P2) 139735.
8. P. Song, J. Yang, C. Wang, T. Wang, H. Gao, G. Wang, J. Li, *Nano-Micro Lett*. **2023**, *15*, 118.
9. Z. Hu, H. Cui, Y. Zhu, G. Lei, Z. Li, *[J. Power Sources](https://www.sciencedirect.com/journal/journal-of-power-sources)* **2022**, *[536](https://www.sciencedirect.com/journal/journal-of-power-sources/vol/536/suppl/C)*, 231438.
10. X. Wu, H. Zhao, J. Xu, Y. Wang, S. Dai, T. Xu, S. Liu, S. Zhang, X. Wang, X. Li, *[J. Alloy. Compd](https://www.sciencedirect.com/journal/journal-of-alloys-and-compounds).* **[2020](https://www.sciencedirect.com/journal/journal-of-alloys-and-compounds/vol/825/suppl/C)**[,](https://www.sciencedirect.com/journal/journal-of-alloys-and-compounds/vol/825/suppl/C) *[825](https://www.sciencedirect.com/journal/journal-of-alloys-and-compounds/vol/825/suppl/C)*, 154173.
